# Polymeric epitope-based vaccine induces protective immunity against group A *Streptococcus*

**DOI:** 10.1038/s41541-023-00695-x

**Published:** 2023-07-14

**Authors:** Shuxiong Chen, Victoria Ozberk, Gayathri Sam, Zennia Jean C. Gonzaga, Ainslie Calcutt, Manisha Pandey, Michael F. Good, Bernd H. A. Rehm

**Affiliations:** 1 Centre for Cell Factories and Biopolymers (CCFB), Griffith Institute for Drug Discovery, Griffith University (Nathan Campus), Nathan, QLD 4111 Australia; 2 The Institute for Glycomics, Griffith University (Gold Coast Campus), Southport, QLD 4215 Australia; 3 Menzies Health Institute Queensland (MHIQ), Griffith University (Gold Coast Campus), Southport, QLD 4215 Australia

**Keywords:** Peptide vaccines, Conjugate vaccines

## Abstract

Group A *Streptococcus* (Strep A) is a life-threatening human pathogen with no licensed vaccine. Here, we used a biopolymer particle (BP) approach to display repeats of Strep A vaccine candidate peptides p*17 and K4S2 derived from M and non-M protein, respectively. BPs densely displaying both peptides (BP-p*17-S2) were successfully assembled in one-step inside an engineered endotoxin-free *Escherichia coli* strain. Purified BP-p*17-S2 showed a spherical core-shell morphology with a biopolymer core and peptide shell. Upon formulation with aluminum hydroxide as adjuvant, BP-p*17-S2 exhibited a mean diameter of 2.9 µm and a positive surface charge of 22 mV. No cytotoxicity was detected when tested against HEK-293 cells. Stability studies showed that BP-p*17-S2 is ambient-temperature stable. Immunized mice showed no adverse reactions, while producing high titers of peptide specific antibodies and cytokines. This immune response could be correlated with protective immunity in an animal model of infection, i.e. intranasal challenge of mice with Strep A, where a significant reduction of >100-fold of pathogen burden in nose-associated lymphoid tissue, lung, and spleen was obtained. The cost-effective scalable manufacture of ambient-temperature stable BPs coated with Strep A peptides combined with their immunogenic properties offer an attractive alternative strategy to current Strep A vaccine development.

## Introduction

Group A *Streptococcus* (Strep A, *S. pyogenes*) is an important global human pathogen that leads to a wide range of infections from such as mild pharyngitis and impetigo to invasive diseases such as toxic shock syndrome, necrotizing fasciitis and cellulitis^1–3^. Strep A pharyngitis has a considerable global health and economic burden^4^. Robust surveillance mechanisms must be put in place to accurately measure and analyze disease burden between populations, and evaluate the potential impact of future Strep A vaccines^5^. This bacterium is heavily equipped with multiple virulence factors and genetic regulators enabling its diverse infection profile^6^. Recurrence of streptococcal infection may result in post-infection sequelae of acute rheumatic fever, rheumatic heart disease, and acute post-streptococcal glomerulonephritis^1,7^. Strep A infections are a major problem in many low- and middle-income countries and indigenous populations of developed countries where inadequate access to health care and socioeconomic issues occur^6^. The main killers of Strep A infection are rheumatic heart disease mostly in resource-poor settings and invasive infection in high-income countries^8^. Excess mortality due to Strep A is indirectly caused by the development of antimicrobial resistance resulting from the massive consumption of antibiotics^9^. Globally, Strep A causes approximately 700 million human infection each year and there are more than 500,000 deaths due to Strep A diseases^1^. Treatments of streptococcal infection are also very expensive^7^. Currently, there is no licensed vaccine available to prevent this infection. Hence, a globally available safe and effective vaccine against Strep A is urgently needed to prevent streptococcal infection to reduce associated morbidity and mortality.

Immunodominant and conserved antigens have been extensively studied and are potential candidates for development of Strep A vaccines. The Strep A M protein, a coiled-coil homodimer surface-embedded protein encoded by the *emm* gene, is one of the major virulence factor produced during Strep A infection^1,10^. The N-terminal epitopes of the M protein are immunodominant, but it is variable and exhibits high sequence viability amongst different serotypes^11^. Nevertheless, C-terminal sequences of the M protein are promising candidates for vaccine development as this region is highly conserved across different Strep A strains and show immunity against homologous and heterologous strains in vaccinated animals^3,12^. In addition, SpyCEP is a highly conserved surface-exposed *S. pyogenes* cell envelope protease. This antigen can inactivate chemokines, compromise neutrophil recruitment, and mediate pathogen dissemination^13^. Disease severity is correlated with overproduction of SpyCEP, which indicates SpyCEP plays an important role in invasive Strep A infection^13,14^. Studies have shown that SpyCEP is a potential vaccine candidate as it is able to induce potent antibody responses that improve vaccine efficacy^13,15^.

Peptides derived from the C-repeat region of the M protein (p*17 with amino acid sequence LRRDLDASREAKNQVERALE) and an epitope of SpyCEP (S2 with amino acid sequence NSDNIKENQFEDFDEDWENF) have been used to develop a soluble peptide-based vaccine candidate which showed protective immunity against the infection in a mouse model^16,17^. The CovR/S system, a two-component regulatory system in Strep A, is involved in the regulation of virulence factors and the expression of genes that are important for bacterial survival in different environments^18^. Chemically synthesized p*17 and S2 conjugated to diphtheria toxin variants, diphtheria toxoid (DT) and cross-reacting material 197 (CRM197), or translationally fused to CRM197, formulated with aluminum hydroxide (alum) showed efficacy in preventing infection by both emm 100, CovR/S wild type and emm 1, CovR/S mutant strain in mice^16,17^. These Strep A soluble peptide-based vaccine candidates represent highly conserved protein regions and hold the promise to induce cross-protection against the various world-wide Strep A strains. Chemical synthesis of peptides, recombinant production of pure immunogenic carrier such as DT or CRM197 and the subsequent chemical conjugation and purification makes these peptide-based conjugate vaccines one of the most expensive vaccines. Hence, in addition to vaccine efficacy it would be desirable that a new vaccine would be ambient-temperature stable and could be cost-effectively produced at scale in order to facilitate world-wide dissemination of the vaccine including in resource-poor settings.

We developed a new vaccine approach that is based on engineering endotoxin-free *Escherichia coli* to assemble spherical biopolymer inclusion which core is composed of the natural non-toxic polymer, polyhydroxybutyrate, and which surface densely displays antigens and epitopes of interest^19–21^. The one-step assembly of biopolymer particles (BPs) coated with antigens/epitopes inside engineered *E. coli* is high-yielding and enables cost-effective scalable manufacture of a synthetic subunit-based particulate vaccine which safety and efficacy against pathogens were previously shown^20,22–31^. Antigen/epitopes displayed on BPs became immunogenic and induced long-lasting, strong and specific antibody and cell-mediated immune responses correlating with protective immunity against different pathogens such as including *Streptococcus suis*^28^, *Mycobacterium tuberculosis*^27,32^, *Plasmodium falciparum*^29^, *SARS-CoV-2*^30^, *Pseudomonas aeruginosa*^33,34^, *Hepatitis C virus*^35^, *Streptococcus pneumoniae*^36^, and *Neisseria meningitidis*^37^. No adverse effects were observed and displayed antigens/epitopes showed superior immunological properties when compared to the corresponding soluble forms^26–28,30,37^.

**Fig. 1:**
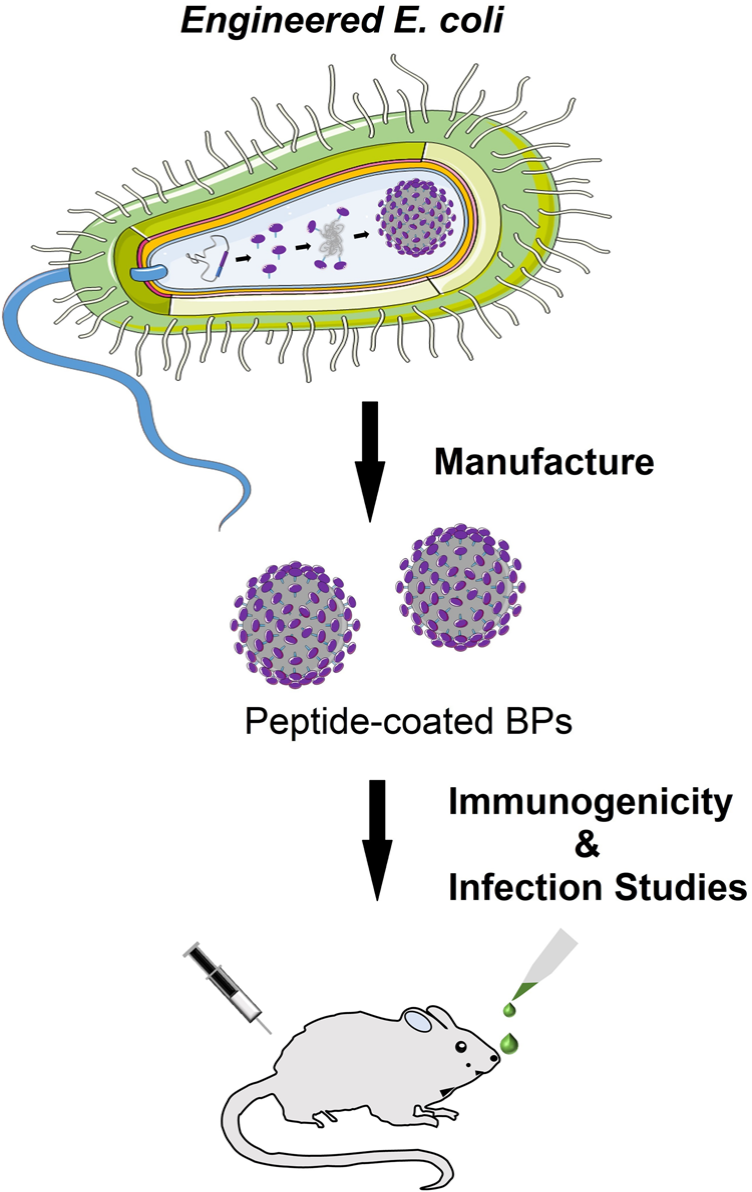
Schematics of BP vaccine manufacture and induction of protective immunity in mouse model. Immunodominant conserved antigens selected from Strep A were used to bioengineer an endotoxin-free *E. coli* strain for production of antigen-coated BP vaccines. Immunogenicity and protective immunity of BP vaccines were evaluated in mouse models.

In this study, the selected conserved and immunodominant Strep A peptides, p*17 and S2, were engineered to densely coat BPs by translationally fusing them to the polyhydroxybutyrate synthase (PhaC) that catalyzes polymer synthesis and mediates BP assembly while remaining attached to its surface (Fig. 1). PhaC performs as anchor to attach antigens/epitopes to the BP surface. The production of BP-p*17-S2 vaccines were carried out in the endotoxin-free *E. coli* strain. Our data presented in this study demonstrated that BPs alone and the epitope-coated BP-p*17-S2 vaccine candidates are not toxic and ambient-temperature stable. Formulation of our BP-p*17-S2 vaccines with alum is able to elicit strong antigen-specific antibody responses and cytokine production, leading to protection against infection by Strep A.

## Results

### Design, production, and characterization of BP-p*17-S2 vaccine candidates

**Fig. 2:**
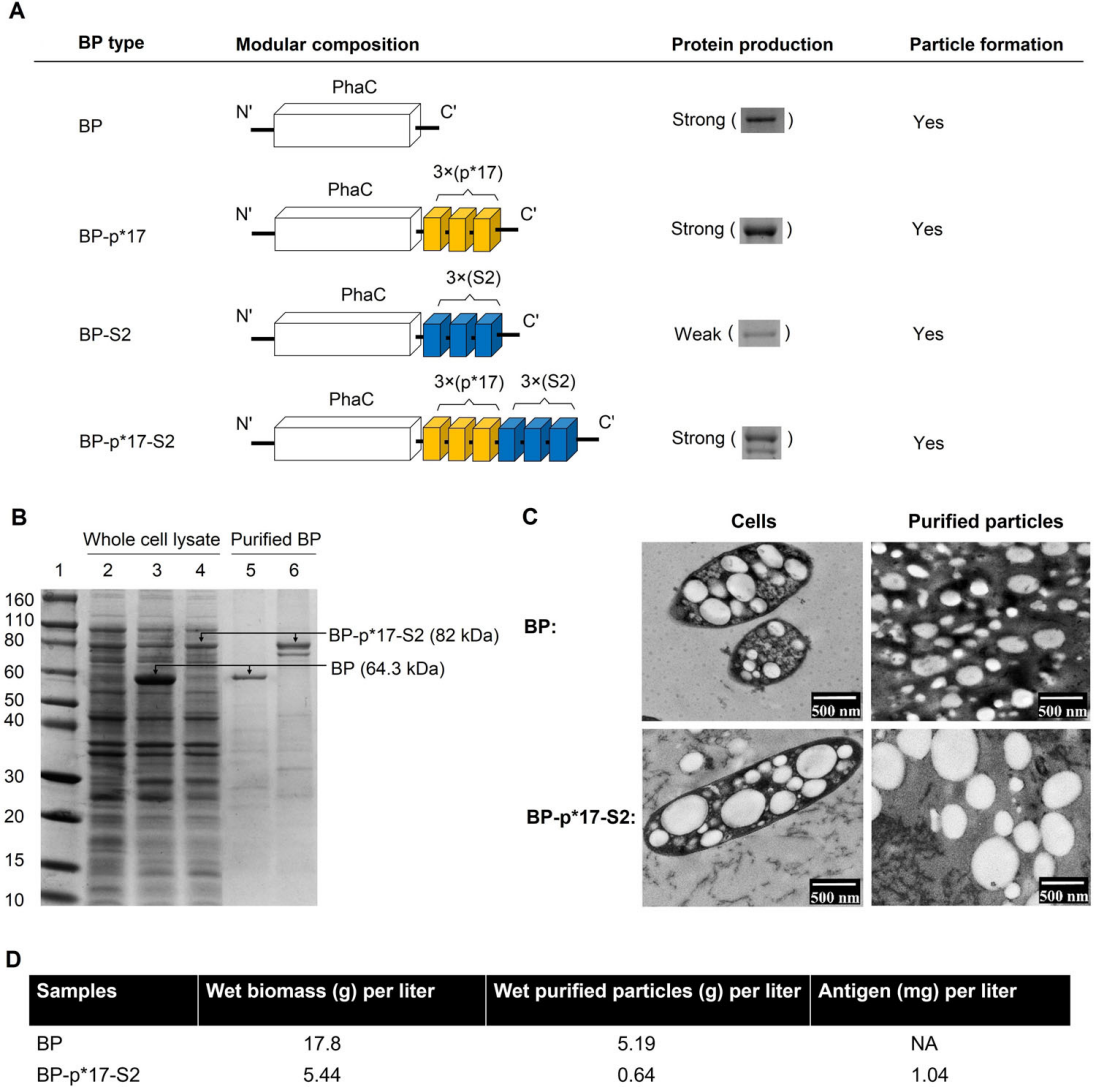
Characterization of BP-p*17-S2 vaccines. **A**. Schematic diagram of recombinant genes encoding fusion proteins that mediated the production of BPs displaying p*17 and/or S2. **B**. Protein profile of purified BP-p*17-S2 vaccine. Lane 1, Molecular weight marker (Novex Sharp Pre-stained Protein Standard, ThermoFisher Scientific); lane 2, *E. coli* alone (negative control); lane 3, *E. coli* producing BPs; lane 4, *E. coli* producing BP-p*17-S2; lane 5, purified BP, 64.3 kDa; lane 6, purified BP-p*17-S2, 82 kDa. **C**. TEM images of *E. coli* producing BP-p*17-S2 vaccines and of purified particles. (Scale bars: 500 nm). **D**. Production yield data.

The modular composition of the hybrid genes encoding the fusion proteins are shown in Fig. 2A. To construct the hybrid genes, the PhaC together with the p*17 and s2 encoding DNA regions were cloned into pET14b expression vector containing the strong T7 promoter^16^. Four plasmid constructs (Supplementary Table 1) were used in this study including the pET14b_PhaC for empty BP production and three plasmids were constructed containing the Strep A antigens fused to the C-terminus of PhaC, pET14b_PhaC-p*17, pET14b_PhaC-S2 and pET14b_PhaC-p*17-S2 to produce BP-p*17, BP-S2 and BP-p*17-S2, respectively. The BP-S2 fusion protein was poorly produced (Fig. 2A), and hence BP-S2 was not included in the final production. In comparison to a mixture of BP-S2 and BP-p*17, BP-p*17-S2 would be the most cost-effective vaccine candidate by producing two epitopes attached to the same BP in a one-step process. Therefore, only empty BP served as negative control and BP-p*17-S2 were used for final production and animal trials.

SDS-PAGE analysis showed the presence of recombinant proteins attached to the BPs and the dominant protein bands corresponded to the theoretical molecular weight (MW) of expected proteins attached to BP (64.3 kDa) and BP-p*17-S2 (82 kDa), respectively (Fig. 2B). The dominant BP-p*17-S2 protein band (top) corresponding to the expected MW of 82 kDa and the directly below but distinct lower MW protein band were excised for Quadrupole time-of-flight mass spectrometry (Q-TOF-MS). The results confirmed the protein sequences of both protein bands in Fig. 2B to match the epitope sequences of BP-p*17-S2 (Supplementary Table 2). However the lower MW protein band N terminus could not be fully detected suggesting a possible cleavage of the N terminus of the PhaC protein. Cells producing BP-p*17-S2 and purified particles were analyzed using TEM (Fig. 2C), which confirmed the production and spherical morphology of BPs. The recombinant proteins were quantified by densitometry (Supplementary Fig. 1). More than 1 mg of purified p*17-S2 antigens was obtained from 1 L cell culture without optimization (Fig. 2D), which demonstrated the high yield manufacture of BP-p*17-S2.

**Fig. 3:**
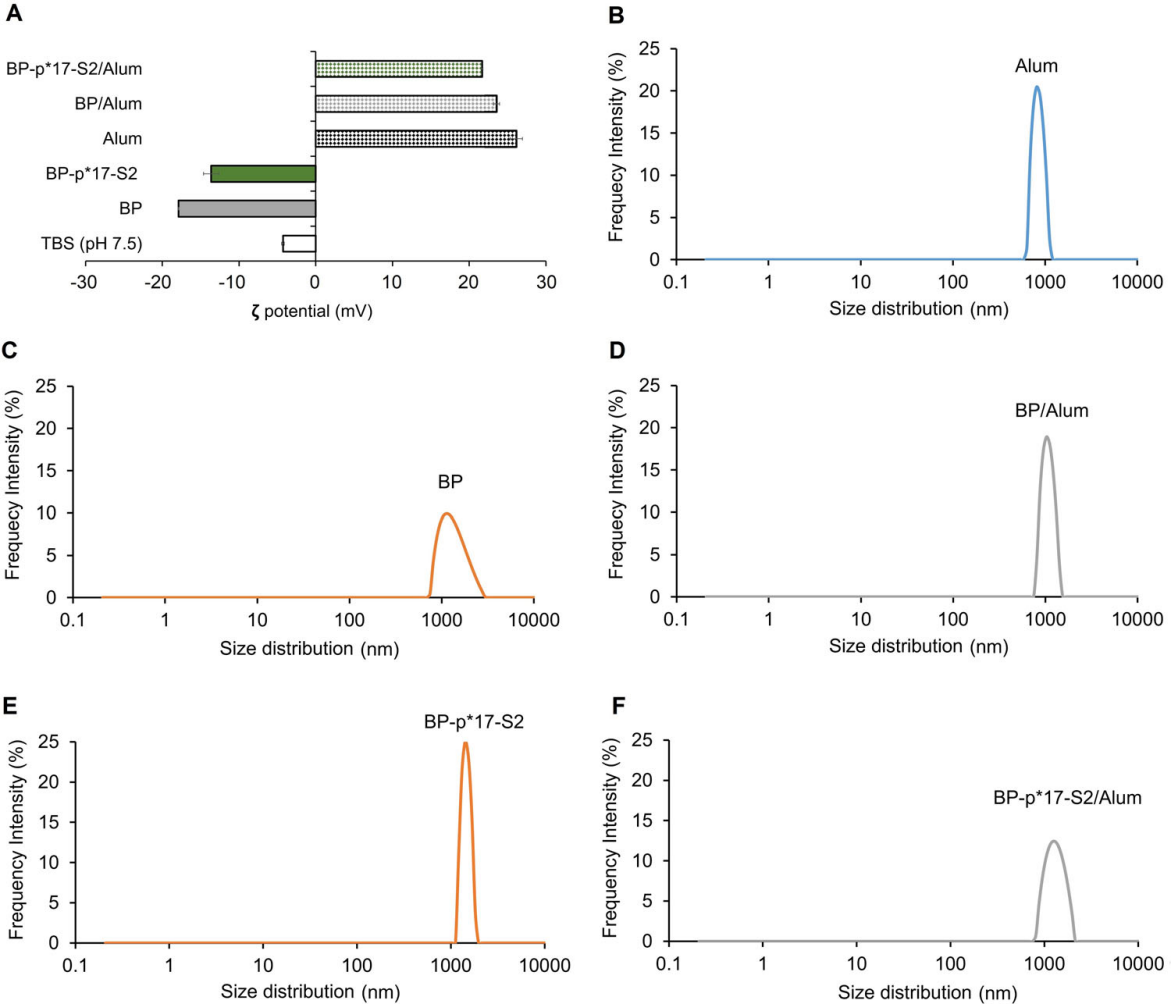
Physicochemical properties of BP-p*17-S2 vaccine candidate. **A**. ζ-potential of BP-p*17-S2 vaccines before and after formulation with alum. **B**. Size distribution of alum. **C, D**. Size distribution of BP before and after formulation with alum. **E, F**. Size distribution of BP-p*17-S2 before and after formulation with alum. All particle size distribution and ζ-potential were consecutively measured 3 times using Litesizer 500 (Anton Paar, Australia). Each data point of measurement represents the mean ± SEM.

Alum was used as the adjuvant in vaccine formulation for animal experiments. In order to analyze the effect of alum on the physiochemical properties of BP-p*17-S2, the size distribution and ζ-potential were measured before and after formulation with alum using Litesizer 500 (Fig. 3). The ζ-potential of empty BPs and BP-p*17-S2 were approximately −17 mV prior to formulation with alum. Alum itself possessed a strongly positively charged surface, 25 mV, which imparted a positive surface charge of about 22 mV to BPs and BP-p*17-S2 upon formulation (Fig. 3A). In addition, size distribution of alum is between 800 nm and 1500 nm (Fig. 3B). The size distribution of BP was narrowed down from 900 nm – 5000 nm to 900 nm – 1800 nm after formulation with alum (Fig. 3C, D). Particle size range of BP-p*17-S2 was similar before and after formulation with alum, which was between 900 nm and 2,900 nm (Fig. 3E, F).

**Fig. 4:**
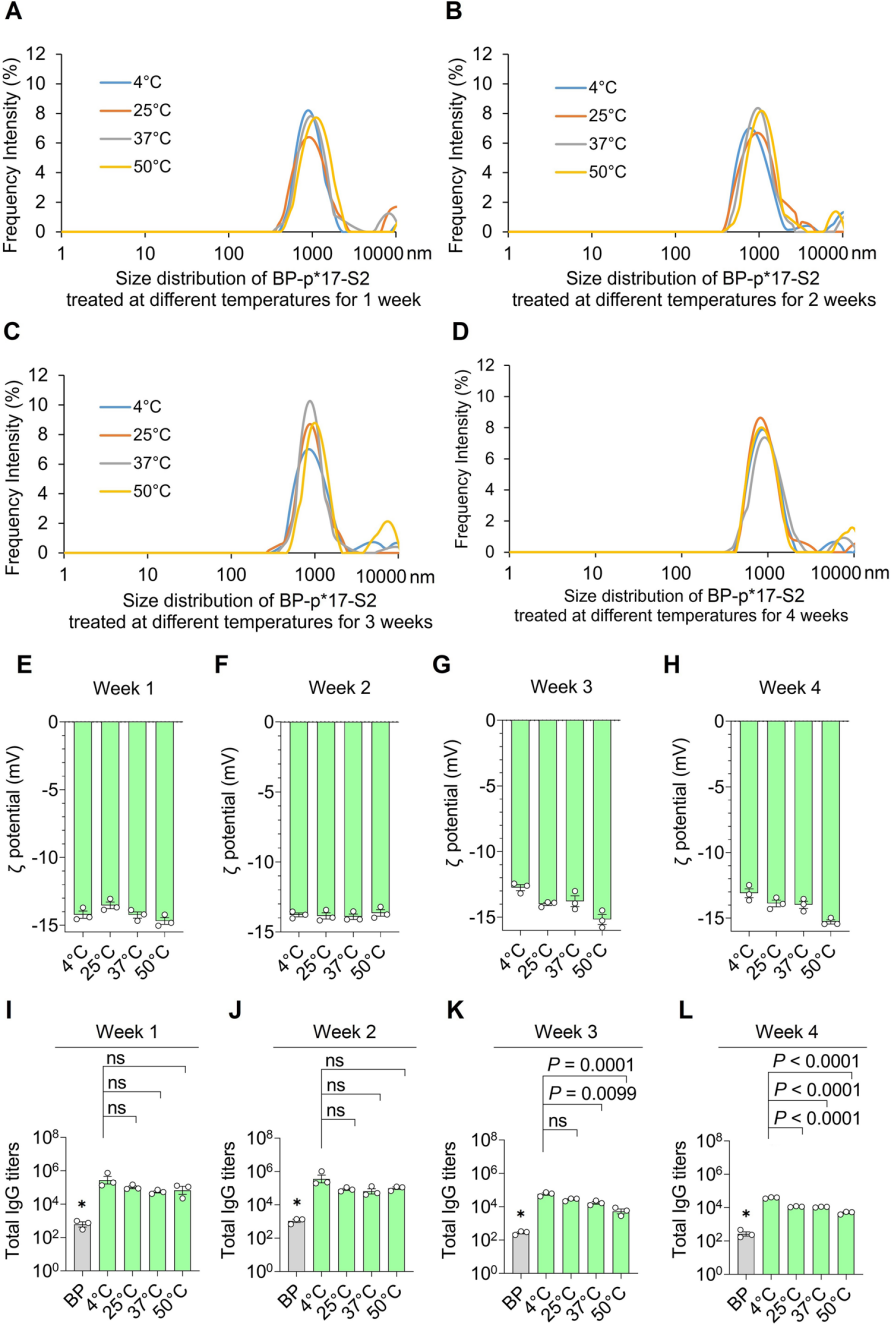
Stability study of BP-p*17-S2 treated at different temperatures for 4 weeks. **A**–**D**. Size distribution of BP-p*17-S2 after different temperature treatments for 1, 2, 3, and 4 weeks. All samples were consecutively measured 3 times using Litesizer 500 (Anton Paar, Australia). Each data point of measurement represents the mean ± SEM. **E**–**H**. ζ-potential of BP-p*17-S2 after treatment with different temperatures for 4 weeks. **I**–**L**. Antigenicity of BP-p*17-S2 after treatment with different temperatures for 4 weeks. This experiment was performed by ELISA using pooled serum samples from mice vaccinated with BP-p*17-S2. n = 3 technical replicates. Means with SEM are plotted. *, statistical significance (*P* values < 0.05). One-way ANOVA Dunnett’s multiple comparisons test was used to compare various temperature-treated vaccines to either BP or BP-p*17-S2 stored at 4 °C.

Ambient-temperature stability is an attractive property of vaccines^16,30^. BP-p*17-S2 vaccine was treated at different temperatures, 4 °C, 25 °C, 37 °C, 50 °C, for 4 weeks. The vaccine stability was investigated by analyzing the protein profile, antigenicity, size distribution and ζ-potential (Fig. 4, Supplementary Fig. 3 and 4). The major size distribution of BP-p*17-S2 remained unchanged, between 900 nm and 2900 nm across different temperature treatments for 4 weeks (Fig. 4A–D). There was no significant difference of the ζ-potential of BP-p*17-S2 treated at different temperatures for 4 weeks (Fig. 4E–H). Sodium dodecyl-sulfate polyacrylamide gel electrophoresis (SDS-PAGE) analysis showed the target protein band of BP-p*17-S2 was stable for 1 week across different temperature treatments and then started to degrade (Supplementary Fig. 3). However, antigenicity of BP-p*17-S2 analyzed using ELISA was not compromised after the incubation at elevated temperatures for 2 weeks (Fig. 4I–L). In addition, the target protein band of BP-p*17-S2 was stable at a given temperature across 4 weeks, and antigenicity of BP-p*17-S2 was not affected across 2 weeks at the given temperatures, 4 °C, 25 °C, 37 °C, 50 °C (Supplementary Fig. 4).

### Immunogenicity study of BP-p*17-S2 using a mouse model

Cytotoxicity of BP-p*17-S2 vaccines was studied before the vaccine was applied for animal studies. HEK-293 has been previously used to analyze the cytotoxicity of nanoparticles^38,39^. The cytotoxicity of empty BPs and BP-p*17-S2 was performed using HEK-293 cell lines (Supplementary Fig. 2). Vaccine particles at the final concentration between 1 and 6% were prepared and incubated with HEK-293 cell lines. Reduction of cell viability was observed in cells treated with different percentage of vaccines; however, there was no significant difference of HEK-293 cell viability between the placebo (cells only) and cells treated with different percentages of BP or BP-p*17-S2 after 24 h incubation. This may indicate that BP and BP-p*17-S2 at concentrations ranging from 1–6% were not toxic to mammalian cells.

**Fig. 5:**
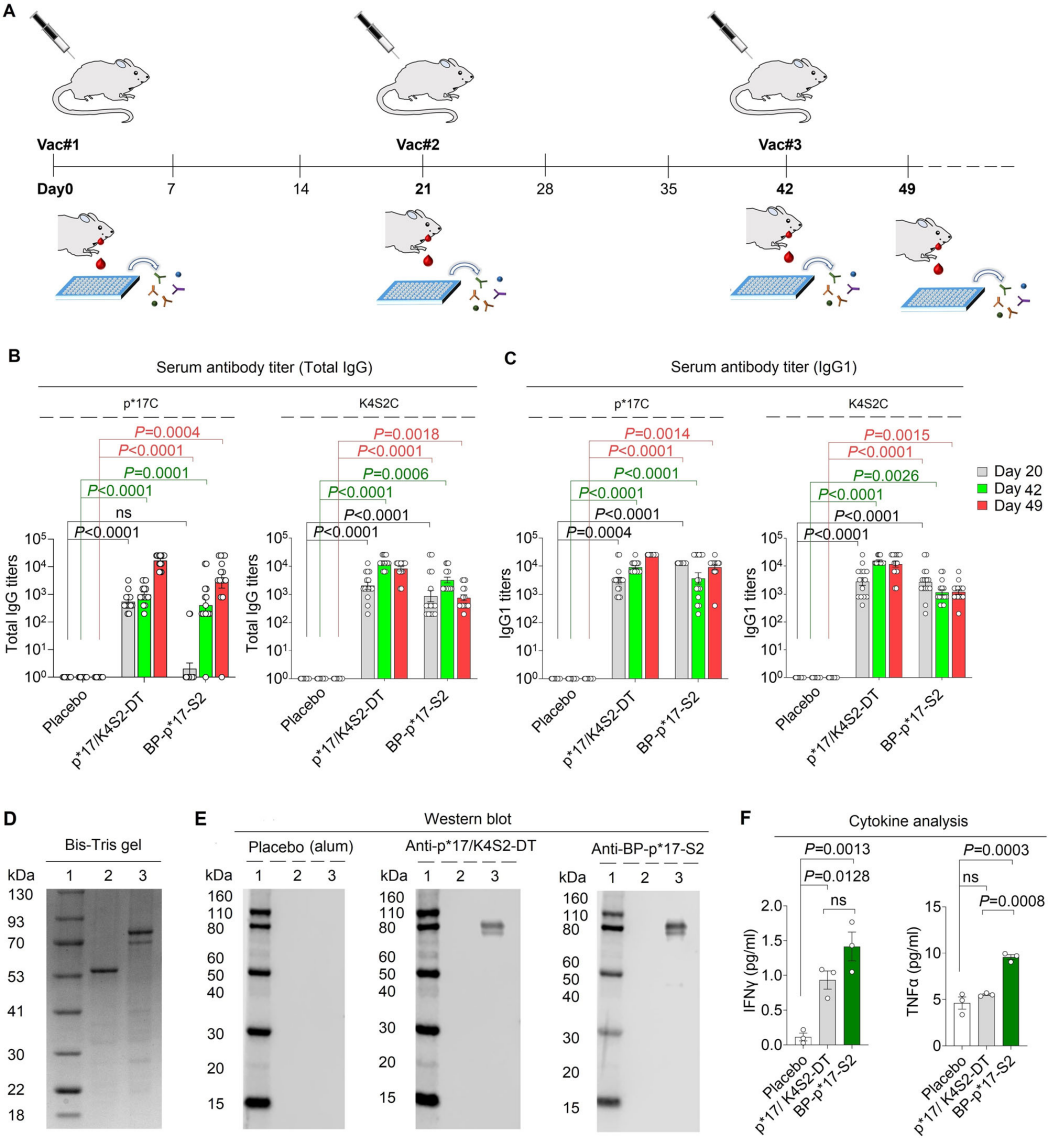
BP-p*17-S2 elicits strong and antigen-specific immune responses. **A**. Schematic diagram of BP-p*17-S2 vaccination plan using female BALB/c mouse model. There are 15 mice per group. **B,** **C**. Mice antibody (total IgG and IgG1) responses to p*17 and K4S2C. n = 15. ns, no significance. Means with SEM and P values < 0.05 are plotted. One-way ANOVA Dunnett’s multiple comparisons test was used to compare immunized to placebo group. **D**. Protein profile of purified BP-p*17-S2 vaccines was analyzed using SDS-PAGE. Lane 1, molecular weight marker (GangNam-Stain pre-stained protein ladder, iNtRon); lane 2, BP, 64.3 kDa; lane 3, BP-p*17-S2, 82 kDa. **E**. Antibody specificity response was evaluated by western blot using pooled serum samples from mice vaccinated with BP, soluble p*17/K4S2-DT, and BP-p*17-S2. Lane 1, molecular weight marker (Novex Sharp Pre-stained Protein Standard, ThermoFisher Scientific); lane 2, BP, 64.3 kDa; lane 3, BP-p*17-S2, 82 kDa. **F**. Cytokine responses from pooled serum samples of mice immunized with various Strep A vaccines. n = 3 technical replicates. ns, no significance. Means with SEM and P < 0.05 are plotted. One-way ANOVA Dunnett’s multiple comparisons test was used to compare vaccinated to placebo groups or soluble p*17/K4S2-DT to placebo/ BP-p*17-S2.

The in vivo safety and immunogenicity of BP-p*17-S2 vaccine candidates were firstly tested in a mouse model (Fig. 5). BALB/c mice were vaccinated intramuscularly (IM) three times with three-weeks interval with alum alone, 5 µg of BP-p*17-S2, and 25 μg of a mixture of the soluble peptides p*17 and K4S2 each conjugated to DT (p*17/K4S2-DT)^17,40^ (Fig. 5A). Alum alone and p*17/K4S2-DT were the negative and positive controls, respectively. Serum samples were collected at defined time-points and ELISAs were performed to determine antibody titers. Specifically, total IgG and IgG1, IgG2a, IgG2b and IgG3 were measured in this study to characterize antigen associated humoral immune responses (Fig. 5B, C and Supplementary Fig. 5). There were no p*17 and S2-specific antibody titers detected in the placebo group, i.e. alum vaccinated mice. For total IgG responses for both p*17 and S2 antigens, the BP-p*17-S2 vaccinated mice induced similar level of antibody responses in comparison to the positive control p*17/K4S2-DT (Fig. 5B). Similar levels of p*17- and S2-specific IgG1 antibody titers were observed in BP-p*17-S2 and p*17/K4S2-DT vaccinated mice (Fig. 5C). IgG2a, IgG2b and IgG3 p*17- and S2-specific antibodies were detectable at similar low levels in groups immunized with BP-p*17-S2 or p*17/K4S2-DT. Serum cytokines from mice immunized with alum, BP-p*17-S2, and p*17/K4S2-DT were analyzed (Fig. 5F). Both BP-p*17-S2 and p*17/K4S2-DT induced significantly higher levels of IFNγ when compared to the placebo group. However, in contrast to the BP-p*17-S2 group, p*17/K4S2-DT did not induce strong TNFα secretion. This result indicated that BP-p*17-S2 induced both strong Th1 (IFNγ) and Th2 (IgG1 and TNFα) type immune responses.

To analyze the antibody response specificity, pooled mice serum samples were used for immunoblotting against various Strep A vaccines (Fig. 5D-E). Pooled serum samples from both BP-p*17-S2 and p*17/K4S2-DT vaccinated mice specifically recognized protein bands corresponding to the theoretical MW BP-p*17-S2 (82 kDa). No bands were detected in the pooled sera from mice vaccinated with alum alone. This result indicated that BP-p*17-S2 induced antigen-specific antibody responses.

### BP-p*17-S2 induce protective immunity against *S. pyogenes* infection

**Fig. 6:**
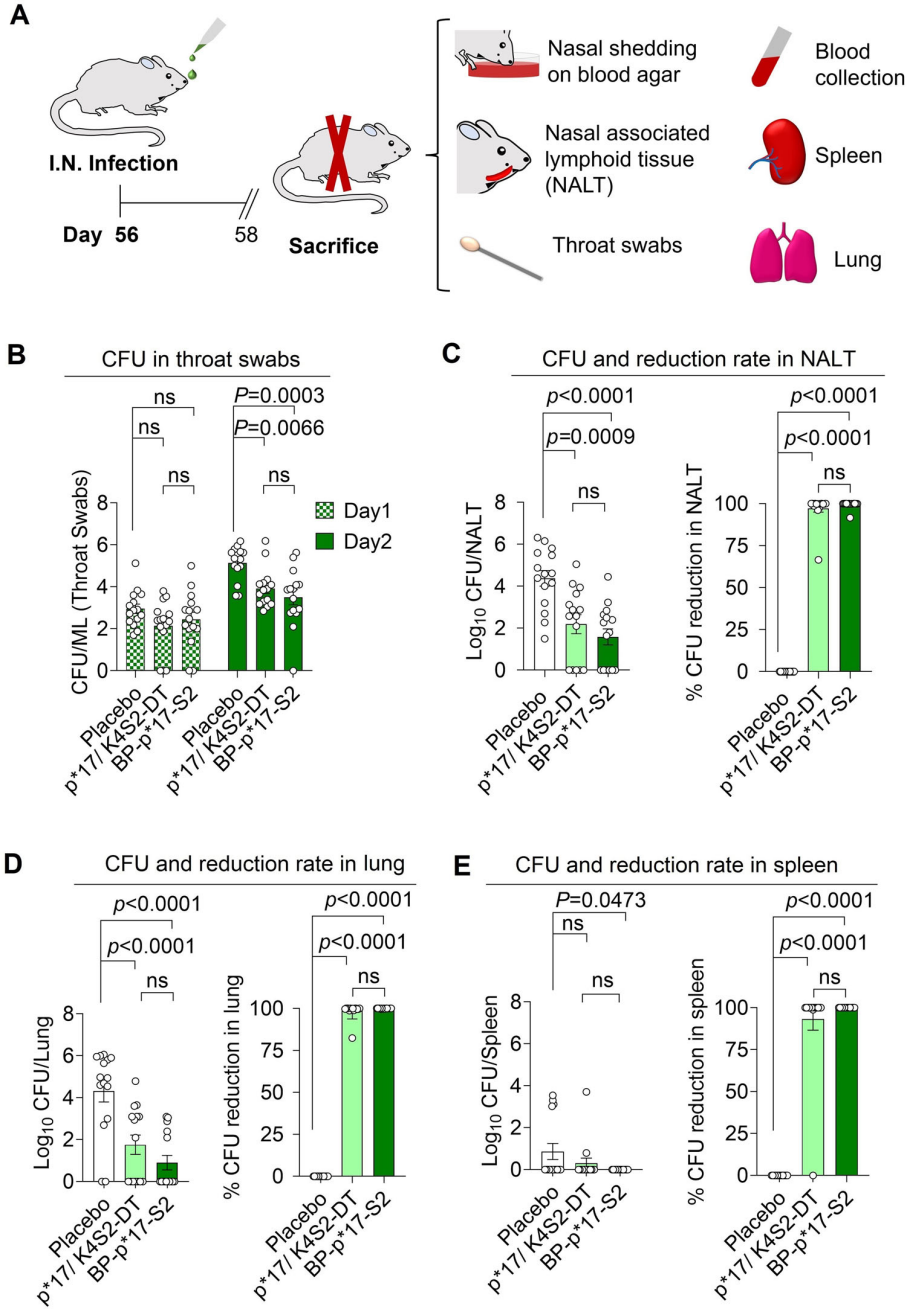
BP-p*17-S2 induces protective immunity in the mouse model of Strep A infection. **A**. Schematic representation of I.N. infection study in BP-p*17-S2 vaccinated female BALB/c mice. There are 15 mice per group. **B**. CFU titer in throat swabs after I.N. challenge. n = 15. ns, no significance. Means with SEM and *P* values < 0.05 are illustrated. One-way ANOVA Dunnett’s multiple comparisons test was used to compare vaccinated to placebo groups or soluble p*17/K4S2-DT to placebo/ BP-p*17-S2. **C**. CFU titer and reduction rate in NALT (Nasal-Associated Lymphoid Tissue) after I.N. infection. N = 15. Ns, no significance. Means with SEM and *P* values < 0.05 are indicated. One-way ANOVA Dunnett’s multiple comparisons test was used to compare immunized to placebo groups or soluble p*17/K4S2-DT to placebo/ BP-p*17-S2. **D**. CFU and reduction rate in lung after I.N. challenge. n = 15. ns, no significance. Means with SEM and *P* values < 0.05 are plotted. One-way ANOVA Dunnett’s multiple comparisons test was used to compare vaccinated to placebo groups or soluble p*17/K4S2-DT to placebo/ BP-p*17-S2. **E**. CFU in spleen after I.N. challenge. n = 15. ns, no significance. Means with SEM and *P* values < 0.05 are indicated. One-way ANOVA Dunnett’s multiple comparisons test was used to compare vaccinated to placebo groups or soluble p*17/K4S2-DT to placebo/ BP-p*17-S2.

Two weeks after the final boost, animals were intranasally (I.N.) infected with 5 × 10^6^ CFUs of Strep A. Nasal shedding was performed on all mice to ensure the successful infection of Strep A (Supplementary Fig. 6A). Mice were active and healthy on day 1 after infection, but some mice developed abnormal behaviors on day 2 (Supplementary Fig. 6 and Supplementary Tables 3–5). All mice were sacrificed on day 2 after the infection (Fig. 6A). Some pathogens were observed in the throats of mice on day 1. However, the pathogen number was significantly reduced in mice vaccinated with BP-p*17-S2 and soluble p*17/K4S2-DT (Fig. 6B). Both vaccine formulations, BP-p*17-S2 and soluble p*17/K4S2-DT, were able to elicit protective immunity against I.N. challenge with Strep A as shown by significant reduction of pathogen number in nasal-associated lymphoid tissue (NALT) and lung (Fig. 6C-D). In addition, I.N. infection with Strep A was invasive as pathogens were observed in the spleen tissue. BP-p*17-S2 vaccination prevented invasiveness of the pathogen as no Step A was detectable in spleens (Fig. 6E).

## Discussion

In this study, two peptides p*17 and S2 were selected for BP-based vaccine development, as they were previously demonstrated to induce strong antibody responses and protective immunity against the invasive streptococcal disease^16,17,30^. Peptide p145 is derived from the conserved carboxyl terminal region of the M protein with amino acid sequence LRRDLDASREAKKQVEKALE^41,42^. Peptide p*17 was developed by substituting the underlined amino acids of p145 peptide sequence to LRRDLDASREAKNQVERALE. This amino acid substitution enhanced p*17 peptide immunogenicity^41^. p*17 showed great stability and induced strong antibody production in a single immunization, leading to 10,000-fold enhanced protection from streptococcal disease^41^. S2 is a highly conserved non-M protein antigen. Additional lysine residues (K4) were added to S2 peptide to improve the K4S2 peptide solubility for soluble conjugate vaccine formulation^17^. K4S2 and p*17 were conjugated to DT and CRM197, respectively, and showed protection from the streptococcal disease in mouse studies^16,17^. Current preparations of Strep A vaccines for human clinical trials are conjugated with CRM197^43^. However, chemical peptide synthesis, recombinant DT or CRM197 manufacture plus chemical conjugation together make these vaccines expensive prohibiting their extensive use and world-wide distribution such as in developing countries. Hence, in order to address these concerns, we applied our recently developed BP vaccine technology to incorporate these peptides for development of a safe particulate subunit vaccine amendable toward cost-effective scalable manufacture using a one-step fermentation process.

The gram-negative bacterium *E. coli* produces endotoxins such as lipopolysaccharides (LPS) found in the outer membrane which are released when cells are lysed^44^. LPS co-purifies with various biological products derived from *E. coli* production strains and causes a wide range of pathophysiological effects, such as systemic inflammation, in both animals and humans^31,44^. To avoid such endotoxic responses triggered by LPS, we used ClearColi BL21 (DE3), an endotoxin-free mutant derived from *E. coli* BL21 (DE3) strain^45^, as the production host to produce BP displaying p*17 and/or S2 vaccines. BP-p*17-S2 has the advantage of the two antigens being produced in a one-step process. Without optimization production of purified BP-p*17-S2 vaccine yielded about 210 vaccine doses per L culture (Fig. 2).

All the vaccines were formulated with alum before vaccination. Addition of alum to BP-p*17-S2 vaccines affected the particle size distribution (Fig. 3), which may be caused by electrostatic interactions within and between BPs and alum. Alum itself is positively charged^27,30^. Alum influenced the shift of the surface charge of the BP-p*17-S2from negative to positive (Fig. 3). The surface charge of the particles in vivo remains unknown. The surface charge of particles may influence the cellular uptake as positively charged are known to be increasingly taken up by dendritic cells^46^. Ambient-temperature stable vaccines hold the promise of facilitated distribution in areas where cold-chain requirement cannot be met. Such vaccines often show extended shelf-life and are easier to stockpile i.e. enhance epidemic preparedness^16^. It was previously demonstrated that proteins attached to BPs retained their functionality at high temperatures such as e.g. the attached carbonic anhydrase remained active after incubation at 90 °C for 1h^47^. In this study, BP-p*17-S2 vaccines were treated at different temperatures for 4 weeks and the results demonstrated retention of antigenicity and physicochemical properties (such as size distribution and ζ-potential) across the different temperatures (Fig. 4, Supplementary Fig. 3 and 4).

Vaccine safety is a crucial component for vaccine development. BP vaccines are composed of a polyhydroxybutyrate core densely coated with selected Strep A antigens, p*17 and S2. Polyhydroxybutyrate itself has been shown to be biocompatible and biodegradable including its approval by the US FDA for uses as tissue engineering material in clinical trials^48^. BP platforms are non-immunogenic and cannot induce detectable immune responses in animals. Hence, BPs are generally well tolerated by mammalian cells^19,24,25,49^. Studies showed in vivo polymer degradation started 3 h after injection of radioactive ^14^C-labeled BP in Wistar rats. The radioactivity decreased slowly and polymer was reduced to about 20% of the initial value after 3 months^49,50^. Mice were vaccinated with 5 µg of BP-p*17-S2 vaccines per dose using a 5% BP suspension which was confirmed not to be cytotoxic when tested with HEK-293 mammalian cells (Supplementary Fig. 2). In addition, the safety of soluble p*17-DT and K4S2-DT was confirmed in a formal toxicology study which led to approval by Health Canada for a Phase 1 Clinical trial^17^. No noticeable abnormal behavior and no adverse effects were observed in mice after vaccination.

Mice were vaccinated with 5 µg of BP-p*17-S2 and 25 µg of p*17/K4S2-DT respectively. A lower BP dose was used informed by previous BP vaccine studies where it could be demonstrated that immunogenicity of peptides/antigens is strongly enhanced when attached to BPs^23,36^. This was also intended to demonstrate the possibility of antigen sparing. The dose of the soluble peptide conjugates was informed by previous studies to ensure sufficient immune responses and protective immunity^17,40^. Both BP-p*17-S2 and p*17/K4S2-DT vaccine formulations induced significant total IgG and IgG1 responses against p*17 C and K4S2C when compared to placebo group. The IgG response was dominated by the IgG1 subclass found to be critical to constitute protective immunity against extracellular pathogens. While both vaccine formulations elicited IFNγ production in serum samples, there was increased IFNγ production induced by BP-p*17-S2 (Fig. 5). A significant TNFα production in serum samples was only induced by BP-p*17-S2 (Fig. 5). IFNγ and TNFα are indicators of cell-mediated and antibody responses, respectively^31,51,52^. Thus suggesting that BP-p*17-S2 can trigger both strong humoral and cellular immunity. Less weight loss was observed in vaccinated mice when compared to unvaccinated groups after the I.N. challenge (Supplementary Fig. 6), indicating the disease severity of the infection caused by Strep A. Mice vaccinated with BP-p*17-S2 and p*17/K4S2-DT vaccines developed protective immunity against I.N. challenge with Strep A as demonstrated by significant reduction of pathogen burden in throat swabs, NALT, and lungs. However, only the BP-p*17-S2 vaccine formulation led to significant reduction of CFUs in the spleen tissue (Fig. 6). This suggested BP-p*17-S2 induce immune responses that directly interfere with Strep A invasiveness and are presumable due to its engagement of additional cell-mediated immunity.

In conclusion, we designed and manufactured the particulate subunit vaccine, BP-p*17-S2, which contains both peptides p*17 and S2 simultaneously tethered to BPs. We developed a streamlined one-step production process using an engineered endotoxin-free *E. coli* strain as production host, altogether underpinning the promise for development of a scalable cost-effective manufacturing process. BP-p*17-S2 vaccines were ambient-temperature stable and induced antigen-specific humoral and cell-mediated immune responses leading to protective immunity against I.N. infection with Strep A. Overall, the here described BP-p*17-S2 preclinical studies demonstrated safety and efficacy while underpinned by an efficient manufacturing process. Next steps would be the clinical development of this vaccine.

## Methods

### Bacterial strains and growth conditions

Bacterial strains, plasmids and primers used in this study are listed in Supplementary Table 1. Primers and pUC57 plasmid DNA were synthesized by Integrated DNA Technologies (IDT) and Biomatik, respectively. *E. coli* XL1-Blue was used as a molecular cloning host, grown at 37 °C in Luria Broth (LB) medium (Difco, Detroit, MI) containing ampicillin (Amp, 100 µg/mL) for pET14b plasmid propagation. ClearColi^TM^ BL21 (DE3) (Lucigen, USA), an endotoxin-free *E. coli* strain, was used for BP production. Approximately 15 mL of overnight cell culture was used to inoculate 500 mL of LB medium supplemented with 0.5% wt/vol NaCl, 1% wt/vol glucose, and appropriate antibiotics (Amp, 100 µg/mL and chloramphenicol (Cm), 50 µg/mL), and incubated at 37 °C at 200 rpm for approximately 3 h. The cell culture was induced by adding isopropyl β-D-1- thiogalactopyranoside (IPTG) (Sigma-Aldrich) at a final concentration of 1 mM when optical density 600 (OD600) reached about 0.5. The cultures were further incubated for BP production at 25 °C for 48 h.

### Plasmid construction for production of BP-Strep A vaccines

The DNA fragments encoding p*17, S2, and p*17-S2 were isolated from pUC57 vector by enzyme digestion with *Xho*I and *BamH*I, followed by fragment separation using agarose gel electrophoresis with GelRed stain (Biotium, USA) and gel purification (New England Biolabs, USA)^25^. The isolated individual inserts were ligated into the linearized pET14b_PhaC vector, generated by *Xho*I and *BamH*I enzyme digestion, using T4 DNA ligase to generate the final plasmids pET14b PhaC-p*17, pET14b PhaC-S2 and pET14b PhaC-p*17-S2. The DNA sequence of final plasmids were confirmed by Griffith University DNA Sequencing Center (Griffith University, Nathan Campus, Australia). The confirmed plasmids were transferred into the endotoxin-free production host, *E. coli* strain ClearColi^TM^ BL21 (DE3) (Lucigen, USA) for production of BPs displaying p*17 and/or S2.

### BPs isolation and purification

The cells producing BP vaccines were harvested by centrifugation at 8000 x g for 15 min at 4 °C and resuspended. Cell sediments were resuspended to 10% cell suspension using lysis buffer (10 mM Tris, 5 mM EDTA and 0.04% w/v SDS, pH 7.5). The cell suspensions were then mechanically disrupted using Microfluidizer M-110P (Microfluidics, USA) at 2000 psi. The BPs were harvested by centrifugation of the disrupted lysate at 8000 x g for 15 min at 4 °C. The isolated BPs were subsequently washed three times by lysis buffer, Triton wash buffer (10 × 10^−3^ M Tris, 5 × 10^−3^ M EDTA, 2% v/v Triton X-100, pH 7.5), and Tris buffer (10 × 10^−3^ M Tris.HCl, pH 7.5). The purified BPs were sterilized with 1 mg/mL Ciprofloxacin and washed three times with Tris buffered saline (TBS) (10 × 10^−3^ M Tris, 150 × 10^−3^ M NaCl, pH 7.5). The sterile BPs vaccines were stored in TBS in 4 °C until vaccine formulation.

### Characterization BP-p*17-S2 vaccines

After isolation and purification of BP-p*17-S2 vaccines, the protein profile of vaccine samples was firstly analyzed by 10% SDS-PAGE. The target recombinant protein bands with theoretical molecular weight were excised, and their amino acid sequences were identified using Q-TOF-MS (Mass Spectrometry Facility, University of Queensland, Australia). Densitometry using bovine serum albumin (BSA) standards ranging between 62.5 ng and 500 ng was used to quantify the target antigen concentrations. The SDS-PAGE images were captured and analyzed using Image Lab Software (Bio-Rad Laboratories, USA). Particles were also characterized using Transmission Electron Microscopy (TEM) to analyze particle morphology and size. Size distribution and ζ-potential of vaccines before and after formulation with alum were measured using Litesizer 500 (Anton Paar, Australia). All the measurements were performed in triplicates.

To investigate the stability of BP-p*17-S2 vaccines, the vaccine samples were treated at different temperatures, 4 °C, 25 °C, 37 °C, and 50 °C, for 4 weeks. The particle size and ζ-potential of various temperature-treated samples were measured using Litesizer 500 (Anton Paar, Australia). Antigenicity of BP-p*17-S2 vaccines treated with different temperatures was also studied using enzyme-linked immunosorbent assay (ELISA) and performed in technical triplicates. Briefly, 100 µL of vaccines at antigen concentration of 5 µg mL^−1^ was coated on high-binding ELISA plates (Greiner Bio-One, Germany) at 4 °C overnight. The plain BP and BP-p*17-S2 stored at 4 °C were used as the negative and positive controls, respectively. BSA solution (3%, wt/vol) prepared with TBS buffer was added plates and incubated for 1 h at 25 °C to block unspecific antibody binding. After three times washes with TBST (10 × 10^−3^ M Tris, 150 × 10^−3^ M NaCl, 0.05% Tween20 pH 7.5), the pooled serum samples from mice immunized with BP-p*17-S2 diluted with TBST from 1/200, to 1/25,600 were used as the primary antibodies to specifically bind to the antigens (p*17 and S2) displayed on BP-p*17-S2 particles and incubated for 1 h at 25 °C. After the plates were washed three times with TBST, goat-anti-mouse IgG-HRP (Abcam, United Kingdom) diluted 1:20,000 with TBST was used as the secondary antibody and incubated at 25 °C for 1 h. After washing with TBST, o-phenylenediamine substrate (OPD) (Sigma-Aldrich, USA) was added to plates and incubated for 15 min at 25 °C. The reaction was stopped by adding 50 µL of 0.5 N H_2_SO_4_ and signal was measured at 490 nm using ELx808iu ultramicrotiter plate reader (Bio-Tek Instruments Inc., USA).

### Cytotoxicity study of BP-p*17-S2 vaccines

HEK-293 cell lines were used to study the cytotoxicity of BP-p*17-S2 vaccines. Briefly, cells were cultured in completed DMEM media supplemented with 10% fetal bovine serum and 0.5% vol/vol Penicillin-Streptomycin solution (ATCC, USA). Cells were seeded per well in sterile 96-well plates at a cell density of 5 × 10^5^ and incubated at 37 °C with 5% CO_2_ for 24 h. After three times washes with PBS buffer (137 × 10^−3^ M NaCl, 2.7 × 10^−3^ M KCl, 10 × 10^−3^ M Na_2_HPO_4_, pH 7.4), different percentages of BP-p*17-S2 vaccines, ranging between 1 and 6%, were added to plates in triplicates and incubate for 24 h. Cells only and cell treated with mefloquinine were used as the negative and positive controls, respectively. Following three times washes with PBS, alamar blue cell viability reagent (ThermoFisher Scientific, USA) was added to the well at the final concentration of 10%. After 8 h incubation, the results were measured at 570 nm and 600 nm with BioTek Synergy H1 Hybrid Microplate reader (Bio-Tek Instruments Inc., USA).

### BP-p*17-S2 vaccine immunization and intranasal infection study

Mice intranasal (I.N.) challenge study was approved by Griffith University Animal Ethics Committee with approval number of GLY/06/21/AEC (Queensland, Australia). Female BALB/c mice (6–8 weeks of age) was used for this experiment and purchased from the Animal Resources Centre (Perth, Australia). There were 15 mice per group, alum, soluble peptide mixture p*17-DT + K4S2-DT (p*17/K4S2-DT), and BP-p*17-S2. The soluble p*17/K4S2-DT and alum alone were the positive and negative controls, respectively. Each dose of formulated BP-p*17-S2 vaccines contained 5 μg of antigens and 25 μL of aluminum hydroxide adjuvant (alum, InvivoGen, USA) prepared in a volume of 50 μL. Formulated soluble p*17/K4S2-DT contained 25 μg of antigens/dose^17^, mixed with 25 μL of alum in the volume of 50 μL.

All mice were vaccinated intramuscularly three times at 3 weeks intervals. Collection of blood sample was performed on day 0, 21, 42, and 49 during immunization. Two weeks after the final immunization, mice were I.N. infected with 5 μL/nare of *S. pyogenes* (covR/S MT strain 5448AP) at a concentration of 5 × 10^8^ CFU per mL. All the mice were anesthetized before infection using intraperitoneal injection of 100 μL ketamine:xylazil: distilled water (1:1:10). Mice were weighed and health status was observed daily after I.N. challenge. Nasal shedding and throat swabs were carried out to confirm bacterial infection. All the mice were sacrificed 2 days later after the challenge. The organ tissues, including the lung, spleen, and nasal-associated lymphoid tissue (NALT) were collected for bacterial burden enumeration to check the protective immunity induced by BP-p*17-S2 vaccines. ${\rm{Reduction}}\; {\rm{rate}}=\left(\frac{{\rm{CFU}}\; {\rm{average}}\; {\rm{in}}\; {\rm{placebo}}-{\rm{CFU}}\; {\rm{in}}\; {\rm{vaccinated}}\; {\rm{individual}}}{{\rm{CFU}}\; {\rm{average}}\; {\rm{in}}\; {\rm{placebo}}}\right)\times 100 \%$

### Enzyme-linked immunosorbent assay

Serum antibody responses were analyzed by ELISA. High-binding plates (Greiner Bio-One, Germany) were coated overnight at 4 °C with 100 µL of 5 µg mL^−1^ of soluble proteins, p*17 C and K4S2C, diluted in TBS buffer. The plates were blocked with 200 µL of 3% BSA in TBS buffer for 1 h at 25 °C. After three times washes with TBST, 100 µL of serially diluted mice serum samples (from 1:200 to 1:25, 600) was used as primary polyclonal antibodies and incubated for 1 h at 25 °C. Plates were washed three times with TBST before the incubation with the secondary antibodies, goat-anti-mouse IgG-HRP, goat-anti-mouse IgG1-HRP, goat-anti-mouse IgG2a-HRP, goat-anti-mouse IgG2b-HRP, and goat-anti-mouse IgG3-HRP (Abcam, United Kingdom), diluted 1:20,000 with TBST at 25 °C for 1 h. After washing three times with TBST, 100 μL of OPD (Sigma-Aldrich, USA) was added on plates and incubated for 15 min at 25 °C and the reaction was stopped using 50 µL of 0.5 N H_2_SO_4_. The results were measured at 490 nm using Elx808iu ultramicrotiter plate reader (Bio-Tek Instruments Inc., USA).

### Immunoblot analysis

The specificity of the antibody responses was investigated using immunoblot. Pooled serum samples from mice vaccinated with different vaccines (alum, p*17/K4S2-DT, and BP-p*17-S2) was used for immunoblot against different Strep A vaccine samples. The protein profile of various purified vaccine samples (BP and BP-p*17-S2) for immunoblot was firstly analyzed and separated by 10% SDS-PAGE. BP was the negative control. The amount of vaccine samples loaded for immunoblot analysis was approximately 50 times less than the amount of samples used for protein profile analysis using SDS-PAGE. Protein samples on SDS-PAGE were transferred to nitrocellulose membrane (ThermoFisher Scientific, USA) using iBlotTM 2 Dry Blotting System (Invitrogen, USA). BSA (3%, wt/vol) was used to incubate with the membrane for 1 h to block unspecific antibody binding. After three times washes with TBST, pooled serum samples (from mice vaccinated with alum, p*17/K4S2-DT, and BP-p*17-S2) diluted 1:2,000 with TBST was used as the primary antibodies and incubated with the membrane for 1 h at 25 °C. Following three times washes with TBST, the membrane was incubated with the secondary antibodies, goat-anti-mouse IgG-HRP, diluted 1:20,000 with TBST for 1 h at 25 °C. After three times washes with TBST, the signal was developed on membrane by incubating with 3 mL of SuperSignal West Pico Stable Peroxide Solution and 3 mL of SuperSignal West Pico Luminol/Enhancer Solution (ThermoFisher Scientific, USA) for 5 min at 25 °C. The blots were imaged using the Odyssey Fc Imaging System (LI-COR Biosciences, USA).

### Cytokine measurement

Measurement of mouse serum cytokines, INFγ and TNFα, was done by using ELISA^16^. Mouse serum INFγ was analyzed using Mouse INFγ Kit (Invitrogen, USA). All the materials were freshly prepared for cytokine measurement and carried out in technical triplicates. Briefly, 100 μL of INFγ standards, ranging between 0.63 and 40 pg mL^−1^, were added to anti-INFγ antibody precoated ELISA plate. Diluted serum samples (20 μL of serum + 80 μL of sample diluent) were added to the plate. Fifty microliters of diluted biotin-conjugated anti-mouse INFγ antibody were added to plates to specifically bind to the mouse serum INFγ captured by the precoated anti- INFγ antibody. The plate was washed with 200 µL of wash buffer after the incubation at 25 °C for 2 h on an orbital shaker at 600 rpm. One hundred microliters of diluted streptavidin-HRP (1:100) using assay buffer was added to plate to bind to biotin-conjugated anti-mouse INFγ antibody and incubated at 25 °C for 30 min on an orbital shaker at 600 rpm. After six times washes with 200 µL of wash buffer, 100 µL of substrate, TMB, was added to plate and incubated at 25 °C for 30 min. One hundred microliters of stop solution was added to stop the reaction and the results were measured at 450 nm using an ELx808iu ultramicrotiter plate reader (Bio-Tek Instruments Inc., USA).

Mouse TNFα High Sensitivity ELISA Kit (Invitrogen, USA) was used to evaluate mouse serum TNFα. Briefly, 100 µL of TNFα standards, ranging between 3.13 and 200 pg mL^−1^, was added to anti-TNFα antibody precoated plate. Diluted mouse serum samples (50 µL of calibrator diluent + 50 µL of serum samples) were prepared and added to plate. Fifty microliters of diluted biotin-conjugated anti-mouse TNFα antibody were added to plates to specifically bind to the mouse serum TNFα captured by the precoated anti- TNFα antibody. The reaction was then incubated at 25 °C for 2 h on an orbital shaker at 600 rpm. After six times washes with 200 µL of wash buffer, 100 µL of diluted streptavidin-HRP (1:100) using assay buffer was added to plate to bind to the biotin-conjugated anti-mouse TNFα antibody and incubated at 25 °C for 1 h on an orbital shaker at 600 rpm. After six times washes with 200 µL of wash buffer, 100 µL of amplification reagent I (Biotinyl-Tyramide) was added to plate and incubated at 25 °C for 15 min on an orbital shaker at 600 rpm. One hundred microliters of amplification reagent II (Streptavidin-HRP) were added to plate and incubated at 25 °C for 30 min on an orbital shaker at 600 rpm following six times washes with 200 µL of wash buffer. After six washes, 100 µL of TMB was added as the substrate and incubated at 25 °C for 30 min. The reaction was stopped by adding 100 µL of stop solution. The absorbance was detected at 450 nm with an ELx808iu ultramicrotiter plate reader (Bio-Tek Instruments Inc., USA).

### Statistical analysis

The data were obtained from a single animal trial. GraphPad Prism 8 (GraphPad Software) was used to analyze all statistics. Statistical significance between two independent groups were compared and determined using two-tailed Mann–Whitney U test. One-way ANOVA Dunnett’s multiple comparisons test was used to analyze the significance of differences across groups. A *P* value < 0.05 was considered statistically significant.

### Reporting summary

Further information on research design is available in the Nature Research Reporting Summary linked to this article.

## Supplementary information

Supplemental Material

REPORTING SUMMARY

## Data Availability

All data from this study are available from the corresponding author upon reasonable request. Plasmids newly generated in this study are available from Addgene with ID numbers.

## Materials availability

Unique biological materials described in this study that are not available from standard commercial resources are available from the authors upon request.

## Code availability

No custom computer code or mathematical algorithm that is deemed central to the conclusions was used in this study.
