# Supplementary material for: Polymeric epitope-based vaccine induces protective immunity against group A Streptococcus
Source: NPJ Vaccines. 2023 Jul 14;8:102. doi: 10.1038/s41541-023-00695-x (PMC10349049; doi:10.1038/s41541-023-00695-x)
Supplement: Supplementary file 1 — Supplemental Material [file 41541_2023_695_MOESM1_ESM.pdf]

Supplementary materials

Supplementary Table 1. Bacterial strains, plasmids and primers used in this study

Strains, Plasmids and Primers	Relevant characteristics	References
**Bacterial strains**		
E. coli		
XL1-Blue	*recA1 endA1 gyrA96 thi-1 hsdR17 supE44 relA1 lac* [F' *proAB lacI^q lacZ* Δ*M15* Tn*10* (Tet^r)]	Stratagene
ClearColi™ BL21(DE3)	F– *ompT hsdSB (rB- mB-) gal dcm lon* λ(DE3 [*lacI lac*UV5-T7 gene 1 *ind1 sam7 nin5*]) *msbA148* Δ*gut*QΔ*kds*D Δ*lpxL*Δ*lpxM*Δ*pagP*Δ*lpxP*Δ*eptA*	Lucigen
**Plasmids**		
pET14b	Amp^r; T7 promoter	Novagen
pMCS69	Cm^r; T7 promoter; pBBR1MCS derivative containing codon optimised genes *phaA* and *phaB* from *C. necator*	[54]
pET14b_PhaC-RV1626	pET-14b_PhaC containing RV1626	[55]
pUC57_p*17	pUC57 derivative containing *E. coli* codon optimized p*17 fragment flanked by *Xho*I/*BamH*I sites	Biomatik
pET14b_PhaC-p*17	Codon optimized enol fragment from pUC57_p*17 inserting into *Xho*I/*BamH*I sites of pET14b_PhaC-RV1626	This study
pUC57_S2	pUC57 derivative containing *E. coli* codon optimized S2 fragment flanked by *Xho*I/*BamH*I sites	Biomatik
pET14b_PhaC-S2	Codon optimized enol fragment from pUC57_S2 inserting into *Xho*I/*BamH*I sites of pET14b_PhaC-RV1626	This study
pUC57_p*17-S2	pUC57 derivative containing *E. coli* codon optimized p*17-S2 fragment flanked by *Xho*I/*BamH*I sites	Biomatik
pET14b_PhaC-p*17-S2	Codon optimized enol fragment from pUC57_p*17-S2 inserting into *Xho*I/*BamH*I sites of pET14b_PhaC-RV1626	This study
pET14b_PhaC	pET-14b containing PhaC	[25]
**Primers**	**Sequence from 5' to 3'**	
C-PhaC forward	AGCCACTGGACTAACGATGC	IDT
T7 reverse	GCTAGTTATTGCTCAGCGG	IDT

Amp^r, ampicillin resistance; Cm^r chloramphenicol resistance

Supplementary Table 2. Q-TOF-MS analysis of proteins attached to the PHB beads

Protein Sequence	Coverage/confirmed peptides
**BP (MW: 64.3 kDa)**	
1 MATGKGAAAS TQEGKSQPFK VTPGPFDPAT WLEWSRQWQG TEGNGHAAAS<br>51 GIPGLDALAG VKIAPAQLGD IQQRYMKDFS ALWQAMAEGK AEATGPLHDR<br>101 RFAGDAWRTN LPYRFAAAFY LLNARALTEL ADAVEADAKT RQRIRFAISQ<br>151 WVDAMSPANF LATNPEAQRL LIESGGESLR AGVRNMMEDL TRGKISQTDE<br>201 SAFEVGRNVA VTEGAVVFEN EYFQLLQYKP LTDKVHARPL LMVPPCINKY<br>251 YILDLQPESS LVRHVVEQGH TVFLVSWRNP DASMAGSTWD DYIEHAAIRA<br>301 IEVARDISGQ DKINVLGFCV GGTIVSTALA VLAARGEHPA ASVTLLTTLL<br>351 DFADTGILDV FVDEGHVQLR EATLGGGAGA PCALLRGLEL ANTFSFLRPN<br>401 DLVWNYVVDN YLKGNTPVPF DLLFWNGDAT NLPGPWYCWY LRHTYLQNEL<br>451 KVPGKLTVCG VPVDLASIDV PTYIYGSRED HIVPWTAAYA STALLANKLR<br>501 FVLGASGHIA GVINPPAKNK RSHWTNDALP ESPQQWLAGA IEHHGSWWPD<br>551 WTAWLAGQAG AKRAAPANYG NARYRAIEPA PGRYVKAKA	79%<br>V21-R74, D78-K90, F115-T140, F146-R180, N185-K234, Y250-R299, D306-R442, L456-K498, F501-K518, R521-562
**BP-p*17-S2 (Top band; MW: 82 kDa)**	
1 MATGKGAAASTQEGKSQPFKVTPGPFDPATWLEWSRQWQGTEGNGHAAAS<br>51 GIPGLDALAGVKIAPAQLGDIQQRYMKDFSALWQAMAEGKAEATGPLHDR<br>101 RFAGDAWRTNLPYRFAAAFYLLNARALTELADAVEADAKTRQRIRFAISQ<br>151 WVDAMSPANFLATNPEAQRLLIESGGESLRAGVRNMMEDLTRGKISQTDE<br>201 SAFEVGRNVAVTEGAVVFENEYFQLLQYKPLTDKVHARPLLMVPPCINKY<br>251 YILDLQPESSLVRHVVEQGHTVFLVSWRNPDASMAGSTWDDYIEHAAIRA<br>301 IEVARDISGQDKINVLGFCVGGTIVSTALAVLAARGEHPAASVTLLTTLL<br>351 DFADTGILDVFVDEGHVQLREATLGGGAGAPCALLRGLELANTFSFLRPN<br>401 DLVWNYVVDNYLKGNTPVPFDLLFWNGDATNLPGPWYCWYLRHTYLQNEL<br>451 KVPGKLTVCGVPVDLASIDVPTYIYGSREDHIVPWTAAYASTALLANKLR<br>501 FVLGASGHIAGVINPPAKNKRSHWTNDALPESPQQWLAGAIEHHGSWWPD<br>551 WTAWLAGQAGAKRAAPANYGNARYRAIEPAPGRYVKAKAVLAVAIDKRGG<br>601 GGG-LINKER-LRRDLDASREAKNQVERALE-LINKER-LRRDLDASREAKNQVER<br>651 ALE-LINKER-LRRDLDASREAKNQVERALE-LINKER-NSDNIKENQFEDFDEDW<br>701 ENF-LINKER-NSDNIKENQFEDFDEDWENF-LINKER-NSDNIKENQFEDFDEDW<br>751 ENF	77%<br>V21-R74, D78-R192, I195-F273, N279-R299, I301-F318, E371-F394, N405-K413, H443-L455, T457-K498, F501-K518, D550-R583, A589-K739
**BP-p*17-S2 (lower MW band)**	
1 MATGKGAAASTQEGKSQPFKVTPGPFDPATWLEWSRQWQGTEGNGHAAAS<br>51 GIPGLDALAGVKIAPAQLGDIQQRYMKDFSALWQAMAEGKAEATGPLHDR<br>101 RFAGDAWRTNLPYRFAAAFYLLNARALTELADAVEADAKTRQRIRFAISQ<br>151 WVDAMSPANFLATNPEAQRLLIESGGESLRAGVRNMMEDLTRGKISQTDE<br>201 SAFEVGRNVAVTEGAVVFENEYFQLLQYKPLTDKVHARPLLMVPPCINKY<br>251 YILDLQPESSLVRHVVEQGHTVFLVSWRNPDASMAGSTWDDYIEHAAIRA<br>301 IEVARDISGQDKINVLGFCVGGTIVSTALAVLAARGEHPAASVTLLTTLL<br>351 DFADTGILDVFVDEGHVQLREATLGGGAGAPCALLRGLELANTFSFLRPN<br>401 DLVWNYVVDNYLKGNTPVPFDLLFWNGDATNLPGPWYCWYLRHTYLQNEL<br>451 KVPGKLTVCGVPVDLASIDVPTYIYGSREDHIVPWTAAYASTALLANKLR<br>501 FVLGASGHIAGVINPPAKNKRSHWTNDALPESPQQWLAGAIEHHGSWWPD<br>551 WTAWLAGQAGAKRAAPANYGNARYRAIEPAPGRYVKAKAVLAVAIDKRGG<br>601 GGG-LINKER-LRRDLDASREAKNQVERALE-LINKER-LRRDLDASREAKNQVER<br>651 ALE-LINKER-LRRDLDASREAKNQVERALE-LINKER-NSDNIKENQFEDFDEDW<br>701 ENF-LINKER-NSDNIKENQFEDFDEDWENF-LINKER-NSDNIKENQFEDFDEDW<br>751 ENF	57%<br>A47-R74, D78-R114, Y120-K139, D152-R192, I195-K234, P239-R263, N279-R299, I301-D306, I324-R335, E371-F394, N405-K413, H443-K455, T486-K498, F501-K518, R563-R573, A576-R583, A589-K689

*Confirmed sequences are in red color.

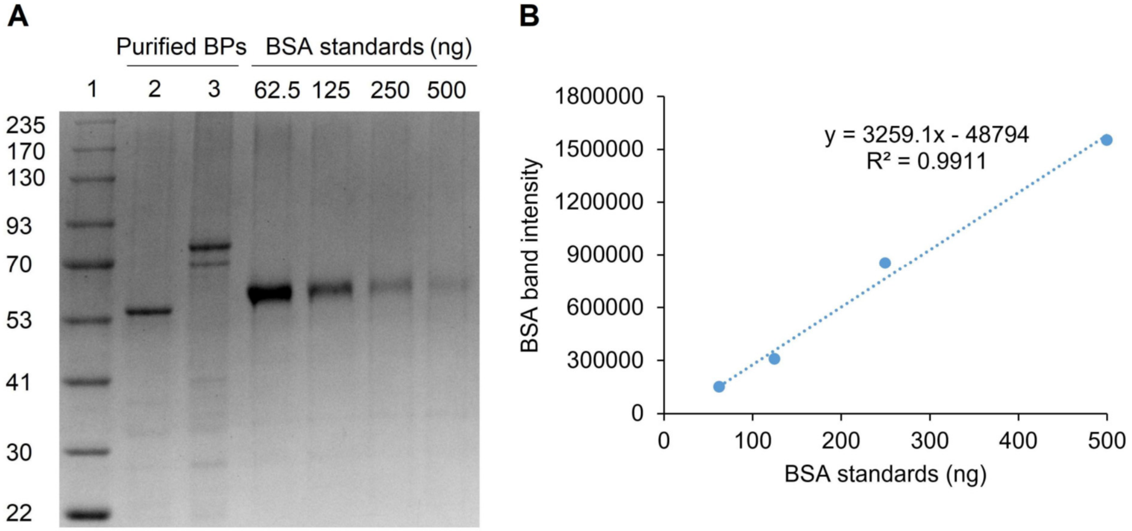

Supplementary Fig. 1 Vaccine concentration calculation. A. Measurement of StrepA antigens (p*17-S2) displayed on the surface of BPs. Lane 1, molecular weight marker (GangNam-Stain prestained protein ladder, iNtRon); lane 2, BP, 64.3 kDa; lane 3, BP-p*17-S2, 82 kDa. **B.** BSA standard curve. BSA, ranging between 62.5 ng and 500 ng, were loaded on 10% Bis-Tris gel to develop a standard curve used to determine the StrepA antigen concentrations in 10% particle suspension. The images were obtained and analyzed respectively by the gel doc (BioRad Laboraties, Hercules, CA) and the Image Lab software (BioRad Laboratories, Hercules, CA).

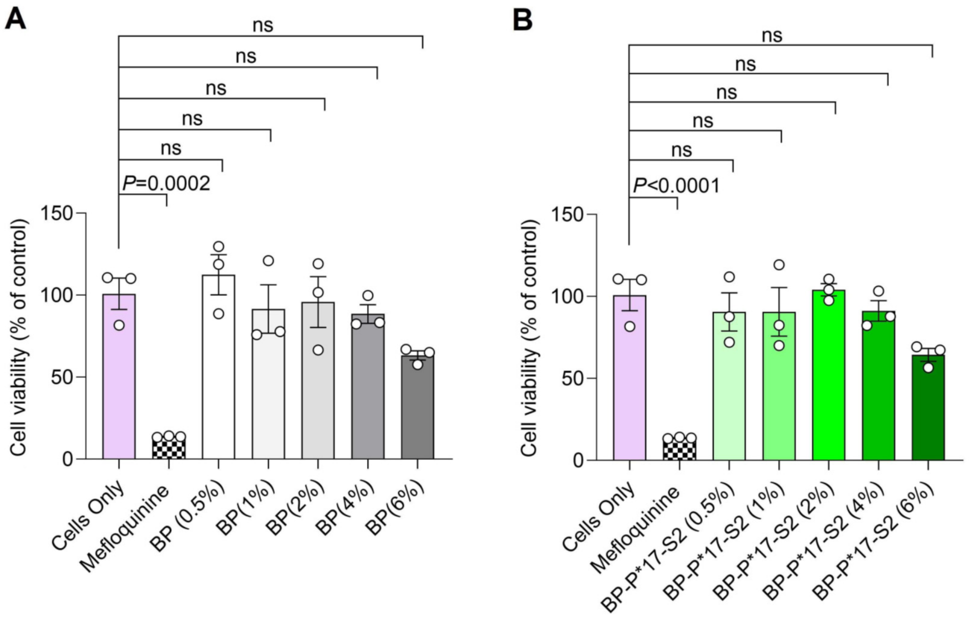

Supplementary Fig. 2 BP-StrepA vaccines cytotoxicity study using HEK-293 cell lines. **A.** BP cytotoxicity analysis. **B.** BP-p*17-S2 cytotoxicity analysis. HEK-293 cell lines were incubated with different percentages of BPs, ranging between 1% and 6%, in triplicates for 24 h. Cell viability was monitored by the reduction of resazurin to resorufin. The results were measured after 8 h incubation with alamar blue. N = 3. ns, no significance. Means with SEM and P values < 0.0005 were indicated. One-way ANOVA Dunnett's multiple comparisons test was used to compare BP-StrepA vaccines and/or melfloquinine-treated to the negative control, cell only group.

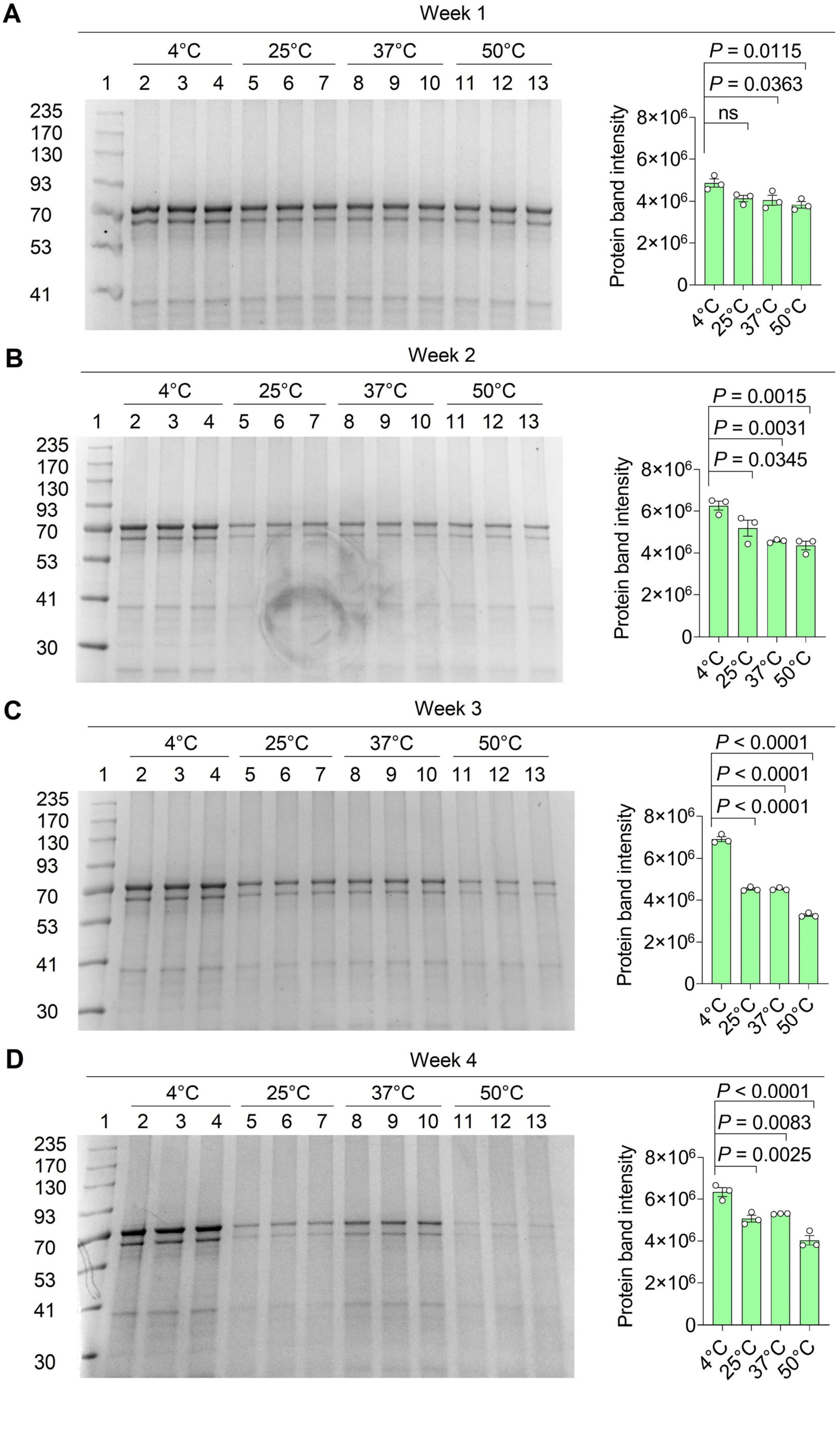

A
Week 1
4°C
25°C
37°C
50°C
1 2 3 4 5 6 7 8 9 10 11 12 13
235
170
130
93
70
53
41
P = 0.0115
P = 0.0363
ns
Protein band intensity
8×10^6
6×10^6
4×10^6
2×10^6
0
4°C 25°C 37°C 50°C
B
Week 2
4°C
25°C
37°C
50°C
1 2 3 4 5 6 7 8 9 10 11 12 13
235
170
130
93
70
53
41
30
P = 0.0015
P = 0.0031
P = 0.0345
Protein band intensity
8×10^6
6×10^6
4×10^6
2×10^6
0
4°C 25°C 37°C 50°C
C
Week 3
4°C
25°C
37°C
50°C
1 2 3 4 5 6 7 8 9 10 11 12 13
235
170
130
93
70
53
41
30
P < 0.0001
P < 0.0001
P < 0.0001
Protein band intensity
8×10^6
6×10^6
4×10^6
2×10^6
0
4°C 25°C 37°C 50°C
D
Week 4
4°C
25°C
37°C
50°C
1 2 3 4 5 6 7 8 9 10 11 12 13
235
170
130
93
70
53
41
30
P < 0.0001
P = 0.0083
P = 0.0025
Protein band intensity
8×10^6
6×10^6
4×10^6
2×10^6
0
4°C 25°C 37°C 50°C

Supplementary Fig. 3 Protein profile of BP-p*17-S2 after incubation at different temperatures for 4 weeks. SDS-PAGE and protein band intensity of sterile BP-p*17-S2 were after incubation at different temperatures for 1 week (A), 2 weeks (B), 3 weeks (C), and 4 weeks (D). Lane 1, molecular weight marker (GangNam-Stain prestained protein ladder, iNtRon); lanes 2-4, BP-p*17-S2 treated at 4 °C; lanes 5-7, BP-p*17-S2 treated at 25 °C; lanes 8-10, BP-p*17-S2 treated at 37 °C; lanes 11-13, BP-p*17-S2 treated at 50 °C. n = 3. Each data point of measurement represents the mean ± SEM. ns, no significance. Means with SEM are plotted. One-way ANOVA Dunnett’s multiple comparisons test was used to compare various temperature-treated vaccines to the vaccine stored at 4 °C.

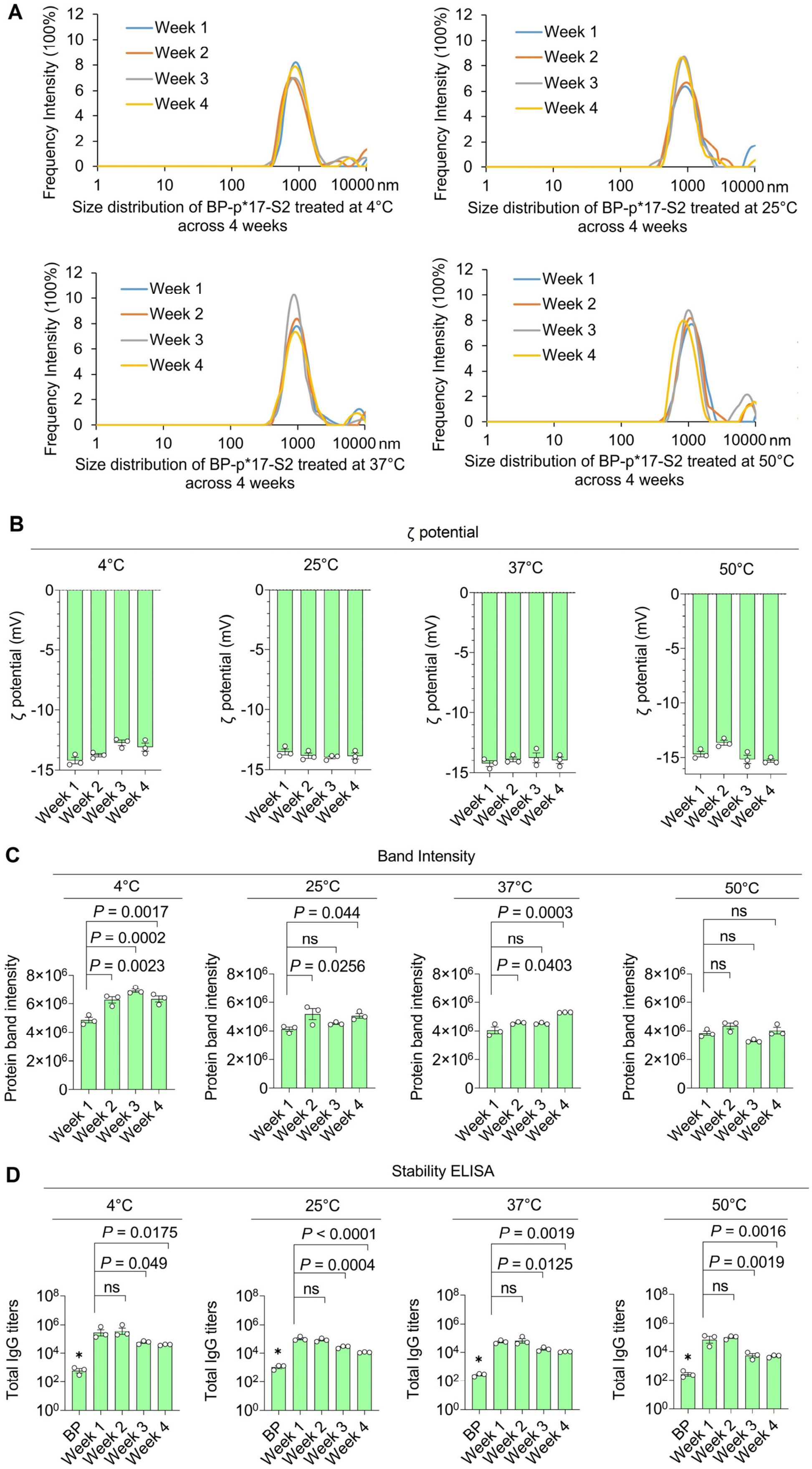

A
Frequency Intensity (100%)
Week 1
Week 2
Week 3
Week 4
nm
Size distribution of BP-p*17-S2 treated at 4°C across 4 weeks
Size distribution of BP-p*17-S2 treated at 25°C across 4 weeks
Size distribution of BP-p*17-S2 treated at 37°C across 4 weeks
Size distribution of BP-p*17-S2 treated at 50°C across 4 weeks
B
ζ potential
4°C
25°C
37°C
50°C
ζ potential (mV)
C
Band Intensity
Protein band intensity
P = 0.0017
P = 0.0002
P = 0.0023
P = 0.044
ns
P = 0.0256
P = 0.0003
ns
P = 0.0403
ns
ns
ns
D
Stability ELISA
Total IgG titers
BP
P = 0.0175
P = 0.049
ns
P < 0.0001
P = 0.0004
ns
P = 0.0019
P = 0.0125
ns
P = 0.0016
P = 0.0019
ns

Supplementary Fig. 4 Characterization of BP-p*17-S2 after incubation at different temperature and times. A. Size distribution of BP-p*17-S2 after different temperature treatments across 4 weeks. All samples were consecutively measured 3 times using Litesizer 500 (Anton Paar, Australia). Each data point of measurement represents the mean ± SEM. **B.** ζ-potential of BP-p*17-S2 after treatment with different temperatures for 4 weeks. All samples were consecutively measured 3 times using Litesizer 500 (Anton Paar, Australia). Each data point of measurement represents the mean ± SEM. **C.** Protein band intensity of BP-p*17-S2 after incubation at different temperatures across 4 weeks. n = 3. Each data point of measurement represents the mean ± SEM. **D.** Antigenicity of BP-p*17-S2 after treatment with different temperatures across 4 weeks. This experiment was performed by ELISA using pooled serum samples from mice vaccinated with BP-p*17-S2. N = 3 technical replicates. Means with SEM are plotted. ns, no significance. *, statistical significance (P values <0.05). One-way ANOVA Dunnett's multiple comparisons test was used to compare various temperature-treated vaccines to either BP or BP-p*17-S2 incubated for one week 1.

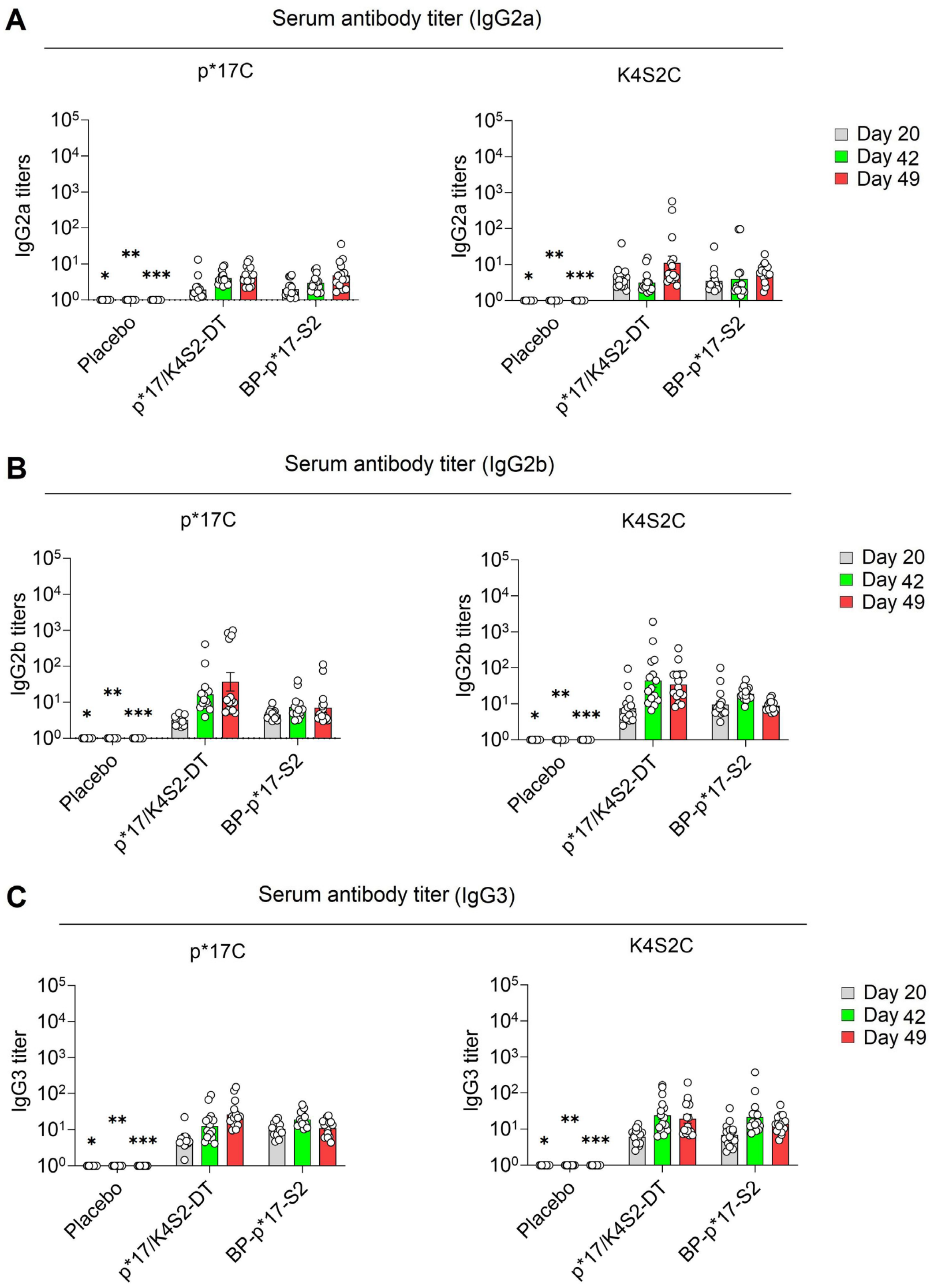

Supplementary Fig. 5 Mice serum antibody analysis. A. IgG2a responses to p*17 and K4S2C. **B.** IgG2b responses to p*17 and K4S2C. **A.** IgG3 responses to p*17 and K4S2C. n = 15. Means with SEM were plotted. One-way ANOVA Dunnett's multiple comparisons test was used to compare immunized to placebo group. * Day 20 serum samples of vaccinated mice were significantly different to placebo ($P < 0.05$). ** Day 42 serum samples of vaccinated mice were significantly different to placebo ($P < 0.05$). *** Day 49 serum samples of vaccinated mice were significantly different to placebo ($P < 0.05$).

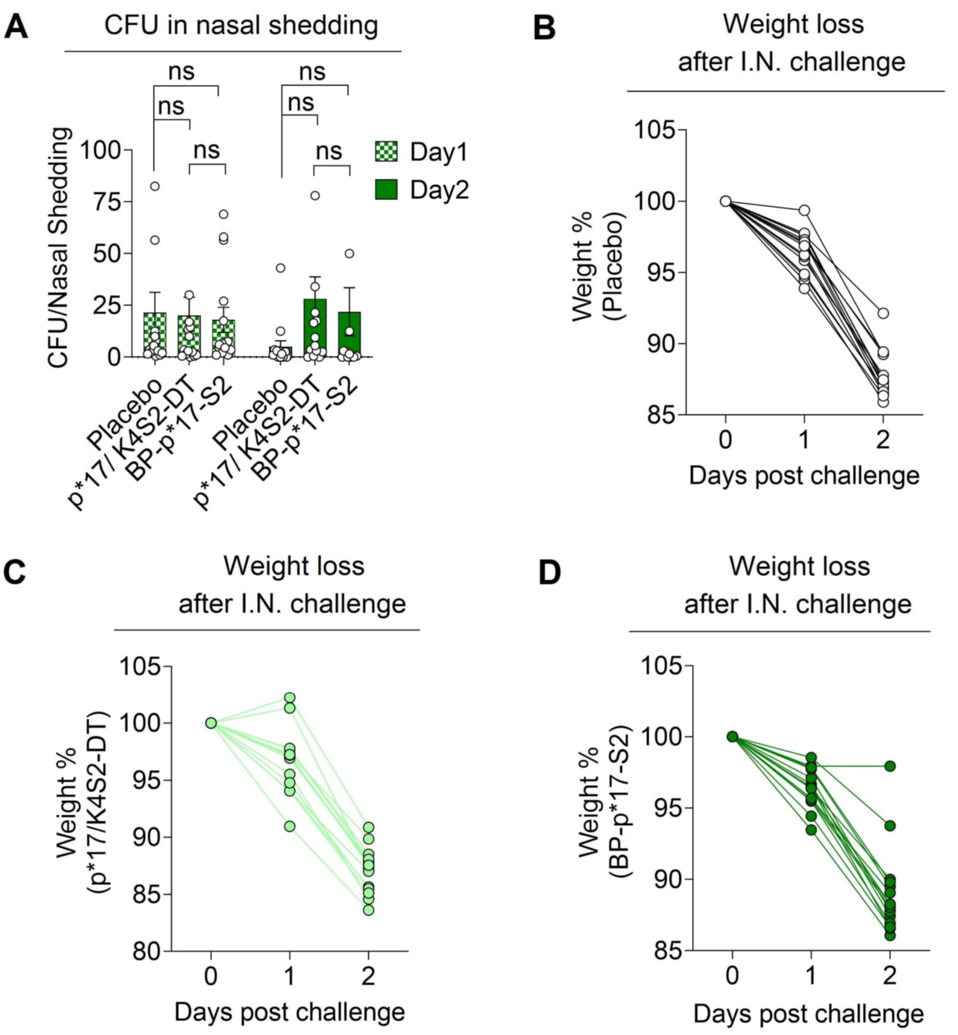

Supplementary Fig. 6 I.N. challenge using female BALB/c mice. A. CFU titre in nasal shedding after I.N. infection. N = 15. ns, no significance. Means with SEM were plotted. ns, no significance. One-way ANOVA Dunnett's multiple comparisons test was used to compare immunized to placebo groups or soluble p*17/K4S2-DT to placebo/ BP-p*17-S2. **B.** Weight change of placebo mice after I.N. infection. **C.** Weight change of p*17/K4S2-DT vaccinated mice after I.N. infection. **D.** Weight change of BP-p*17-S2 vaccinated mice after I.N. infection.

Supplementary Table 3. Scoresheet for monitoring placebo mice undergoing procedure after I.N. challenge

	**Day 1**					**Day 2**				
**Appearance**	1: 1<br>6: 0<br>11: 0	2: 1<br>7: 0<br>12: 0	3: 1<br>8: 1<br>13: 1	4: 1<br>9: 0<br>14: 0	5: 1<br>10: 0<br>15: 0	1: 2<br>6: 0<br>11: 2	2: 2<br>7: 2<br>12: 2	3: 2<br>8: 1<br>13: 2	4: 2<br>9: 2<br>14: 2	5: 2<br>10: 2<br>15: 2
**Hunched posture**	1: 0<br>6: 0<br>11: 0	2: 0<br>7: 0<br>12: 0	3: 0<br>8: 0<br>13: 0	4: 0<br>9: 0<br>14: 0	5: 0<br>10: 0<br>15: 0	1: 0<br>6: 0<br>11: 0	2: 0<br>7: 0<br>12: 0	3: 0<br>8: 0<br>13: 0	4: 0<br>9: 0<br>14: 0	5: 0<br>10: 0<br>15: 0
**Response to stimulus**	1: 0<br>6: 0<br>11: 0	2: 0<br>7: 0<br>12: 0	3: 0<br>8: 0<br>13: 0	4: 0<br>9: 0<br>14: 0	5: 0<br>10: 0<br>15: 0	1: 0<br>6: 0<br>11: 0	2: 0<br>7: 0<br>12: 0	3: 0<br>8: 0<br>13: 0	4: 0<br>9: 0<br>14: 0	5: 0<br>10: 0<br>15: 0
**Level of activity**	1: 0<br>6: 0<br>11: 0	2: 0<br>7: 0<br>12: 0	3: 0<br>8: 0<br>13: 0	4: 0<br>9: 0<br>14: 0	5: 0<br>10: 0<br>15: 0	1: 0<br>6: 0<br>11: 0	2: 0<br>7: 0<br>12: 0	3: 0<br>8: 0<br>13: 0	4: 0<br>9: 0<br>14: 0	5: 0<br>10: 0<br>15: 0
**Eyes**	1: 0<br>6: 0<br>11: 0	2: 0<br>7: 0<br>12: 0	3: 0<br>8: 0<br>13: 0	4: 0<br>9: 0<br>14: 0	5: 0<br>10: 0<br>15: 0	1: 1<br>6: 0<br>11: 0	2: 0<br>7: 0<br>12: 0	3: 0<br>8: 0<br>13: 1	4: 0<br>9: 1<br>14: 0	5: 0<br>10: 0<br>15: 0
**Blood in throat swabs**	1: 0<br>6: 0<br>11: 0	2: 0<br>7: 0<br>12: 0	3: 0<br>8: 0<br>13: 0	4: 0<br>9: 0<br>14: 0	5: 0<br>10: 0<br>15: 0	1: 0<br>6: 0<br>11: 0	2: 0<br>7: 0<br>12: 0	3: 0<br>8: 0<br>13: 0	4: 0<br>9: 1<br>14: 0	5: 0<br>10: 0<br>15: 1
**Weight loss**	1: 0<br>6: 1<br>11: 0	2: 0<br>7: 0<br>12: 0	3: 1<br>8: 1<br>13: 0	4: 0<br>9: 0<br>14: 1	5: 0<br>10: 0<br>15: 0	1: 2<br>6: 2<br>11: 2	2: 2<br>7: 1<br>12: 2	3: 2<br>8: 2<br>13: 2	4: 2<br>9: 2<br>14: 2	5: 2<br>10: 2<br>15: 2

Appearance: 0, coat is smooth; 1, patches of hair piloerected; 2, majority of back is piloerected; 3, piloerection present (mouse appears puffy).

Hunched posture: 0, normal posture; 1, slightly hunched; 2, moderately hunched.

Response to stimulus: 0, mouse responds immediately to auditory stimulus or touch; 1, slow or no response to auditory stimulus, strong response to touch-moves to escape; 2, no response to auditory stimulus, moderate response to touch-moves a few steps.

Level of activity: 0, mouse is active (eating, drinking, climbing, running, fighting); 1, mouse avoids standing upright but the mouse is moving around the bottom of cage; 2, mouse activity is slowed/suppressed with occasional investigative movements.

Eyes: 0, open; 1, eyes not fully open, possibly with secretions; 2, eyes at least half closed, possibly with secretion; 3, eyes closed or milky.

Blood in throat swabs: 0, no; 1, yes (mild); 2, yes, (severe).

Weight loss: 0, 0 to 5% weight loss; 1, 5 to 10% weight loss; 2, 10 to 15% weight loss.

Supplementary Table 4. Scoresheet for monitoring p*17/K4S2-DT vaccinated mice undergoing procedure after I.N. challenge

	Day 1					Day 2				
**Appearance**	1: 0<br>6: 0<br>11: 1	2: 1<br>7: 0<br>12: 1	3: 0<br>8: 1<br>13: 0	4: 0<br>9: 1<br>14: 1	5: 0<br>10: 1<br>15: 0	1: 2<br>6: 2<br>11: 1	2: 2<br>7: 2<br>12: 2	3: 2<br>8: 2<br>13: 2	4: 2<br>9: 2<br>14: 2	5: 2<br>10: 2<br>15: 2
**Hunched posture**	1: 0<br>6: 0<br>11: 0	2: 0<br>7: 0<br>12: 0	3: 0<br>8: 0<br>13: 0	4: 0<br>9: 0<br>14: 0	5: 0<br>10: 0<br>15: 0	1: 0<br>6: 0<br>11: 0	2: 0<br>7: 0<br>12: 0	3: 0<br>8: 0<br>13: 0	4: 0<br>9: 0<br>14: 0	5: 0<br>10: 0<br>15: 0
**Response to stimulus**	1: 0<br>6:<br>11: 0	2: 0<br>7:<br>12: 0	3: 0<br>8:<br>13: 0	4: 0<br>9:<br>14: 0	5: 0<br>10:<br>15: 1	1: 0<br>6:<br>11: 0	2: 0<br>7:<br>12: 0	3: 0<br>8:<br>13: 0	4: 0<br>9:<br>14: 0	5: 0<br>10:<br>15: 0
**Level of activity**	1: 0<br>6: 0<br>11: 0	2: 0<br>7: 0<br>12: 0	3: 0<br>8: 0<br>13: 0	4: 0<br>9: 0<br>14: 0	5: 0<br>10: 0<br>15: 0	1: 0<br>6: 0<br>11: 0	2: 0<br>7: 0<br>12: 0	3: 0<br>8: 0<br>13: 0	4: 0<br>9: 0<br>14: 0	5: 0<br>10: 0<br>15: 0
**Eyes**	1: 0<br>6:<br>11: 0	2: 0<br>7:<br>12: 0	3: 0<br>8:<br>13: 0	4: 0<br>9:<br>14: 0	5: 0<br>10:<br>15: 0	1: 0<br>6:<br>11: 0	2: 0<br>7:<br>12: 0	3: 0<br>8:<br>13: 1	4: 0<br>9:<br>14: 0	5: 1<br>10:<br>15: 0
**Blood in throat swabs**	1: 0<br>6: 0<br>11: 0	2: 0<br>7: 0<br>12: 0	3: 0<br>8: 0<br>13: 0	4: 0<br>9: 0<br>14: 0	5: 0<br>10: 0<br>15: 0	1: 0<br>6: 0<br>11: 0	2: 0<br>7: 0<br>12: 0	3: 0<br>8: 0<br>13: 1	4: 0<br>9: 0<br>14: 0	5: 0<br>10: 0<br>15: 0
**Weight loss**	1: 0<br>6: 1<br>11: 0	2: 0<br>7: 0<br>12: 1	3: 1<br>8: 0<br>13: 0	4: 0<br>9: 0<br>14: 0	5: 0<br>10: 0<br>15: 0	1: 2<br>6: 2<br>11: 2	2: 2<br>7: 2<br>12: 2	3: 2<br>8: 2<br>13: 2	4: 2<br>9: 1<br>14: 2	5: 2<br>10: 2<br>15: 2

Appearance: 0, coat is smooth; 1, patches of hair piloerected; 2, majority of back is piloerected; 3, piloerection present (mouse appears puffy).

Hunched posture: 0, normal posture; 1, slightly hunched; 2, moderately hunched.

Response to stimulus: 0, mouse responds immediately to auditory stimulus or touch; 1, slow or no response to auditory stimulus, strong response to touch-moves to escape; 2, no response to auditory stimulus, moderate response to touch-moves a few steps.

Level of activity: 0, mouse is active (eating, drinking, climbing, running, fighting); 1, mouse avoids standing upright but the mouse is moving around the bottom of cage; 2, mouse activity is slowed/suppressed with occasional investigative movements.

Eyes: 0, open; 1, eyes not fully open, possibly with secretions; 2, eyes at least half closed, possibly with secretion; 3, eyes closed or milky.

Blood in throat swabs: 0, no; 1, yes (mild); 2, yes, (severe).

Weight loss: 0, 0 to 5% weight loss; 1, 5 to 10% weight loss; 2, 10 to 15% weight loss.

Supplementary Table 5. Scoresheet for monitoring BP-p*17-S2 vaccinated mice undergoing procedure after I.N. challenge

	Day 1					Day 2				
**Appearance**	1: 0	2: 1	3: 1	4: 1	5: 1	1: 2	2: 2	3: 2	4: 2	5: 2
	6: 0	7: 1	8: 0	9: 1	10: 0	6: 1	7: 1	8: 2	9: 2	10: 2
	11: 0	12: 0	13: 1	14: 1	15: 1	11: 2	12: 2	13: 2	14: 1	15: 2
**Hunched posture**	1: 0	2: 0	3: 0	4: 0	5: 0	1: 0	2: 0	3: 0	4: 0	5: 0
	6: 0	7: 0	8: 0	9: 0	10: 0	6: 0	7: 0	8: 0	9: 0	10: 0
	11: 0	12: 0	13: 0	14: 0	15: 0	11: 0	12: 0	13: 0	14: 0	15: 0
**Response to stimulus**	1: 0	2: 0	3: 0	4: 0	5: 0	1: 0	2: 0	3: 0	4: 0	5: 0
	6: 0	7: 0	8: 0	9: 0	10: 0	6: 0	7: 0	8: 0	9: 0	10: 0
	11: 0	12: 0	13: 0	14: 0	15: 0	11: 0	12: 0	13: 0	14: 0	15: 0
**Level of activity**	1: 0	2: 0	3: 0	4: 0	5: 0	1: 0	2: 0	3: 0	4: 0	5: 0
	6: 0	7: 0	8: 0	9: 0	10: 0	6: 0	7: 0	8: 0	9: 0	10: 0
	11: 0	12: 0	13: 0	14: 0	15: 0	11: 0	12: 0	13: 0	14: 0	15: 0
**Eyes**	1: 0	2: 0	3: 0	4: 0	5: 0	1: 0	2: 0	3: 0	4: 0	5: 0
	6: 0	7: 0	8: 0	9: 0	10: 0	6: 0	7: 0	8: 0	9: 0	10: 0
	11: 0	12: 0	13: 0	14: 0	15: 0	11: 0	12: 0	13: 0	14: 0	15: 0
**Blood in throat swabs**	1: 0	2: 0	3: 0	4: 0	5: 0	1: 0	2: 0	3: 0	4: 0	5: 0
	6: 0	7: 0	8: 2	9: 0	10: 0	6: 0	7: 0	8: 0	9: 0	10: 0
	11: 0	12: 0	13: 0	14: 0	15: 0	11: 0	12: 0	13: 0	14: 0	15: 0
**Weight loss**	1: 0	2: 0	3: 0	4: 1	5: 0	1: 2	2: 1	3: 0	4: 2	5: 2
	6: 1	7: 0	8: 2	9: 0	10: 0	6: 2	7: 2	8: 2	9: 2	10: 2
	11: 0	12: 0	13: 0	14: 0	15: 1	11: 2	12: 2	13: 2	14: 1	15: 2

Appearance: 0, coat is smooth; 1, patches of hair piloerected; 2, majority of back is piloerected; 3, piloerection present (mouse appears puffy).

Hunched posture: 0, normal posture; 1, slightly hunched; 2, moderately hunched.

Response to stimulus: 0, mouse responds immediately to auditory stimulus or touch; 1, slow or no response to auditory stimulus, strong response to touch-moves to escape; 2, no response to auditory stimulus, moderate response to touch-moves a few steps.

Level of activity: 0, mouse is active (eating, drinking, climbing, running, fighting); 1, mouse avoids standing upright but the mouse is moving around the bottom of cage; 2, mouse activity is slowed/suppressed with occasional investigative movements.

Eyes: 0, open; 1, eyes not fully open, possibly with secretions; 2, eyes at least half closed, possibly with secretion; 3, eyes closed or milky.

Blood in throat swabs: 0, no; 1, yes (mild); 2, yes, (severe).

Weight loss: 0, 0 to 5% weight loss; 1, 5 to 10% weight loss; 2, 10 to 15% weight loss.
